# Association of major blood lipids with post‐stroke dementia: A community‐based cohort study

**DOI:** 10.1111/ene.15219

**Published:** 2022-01-26

**Authors:** Zhirong Yang, Duncan Edwards, Stephen Burgess, Carol Brayne, Jonathan Mant

**Affiliations:** ^1^ Primary Care Unit Department of Public Health and Primary Care School of Clinical Medicine University of Cambridge Cambridge UK; ^2^ Shenzhen Institute of Advanced Technology Chinese Academy of Sciences Shenzhen China; ^3^ MRC Biostatistics Unit School of Clinical Medicine University of Cambridge Cambridge UK; ^4^ Cardiovascular Epidemiology Unit Department of Public Health and Primary Care School of Clinical Medicine University of Cambridge Cambridge UK; ^5^ Cambridge Public Health School of Clinical Medicine University of Cambridge Cambridge UK

**Keywords:** dementia, HDL cholesterol, LDL cholesterol, stroke, triglycerides

## Abstract

**Background and purpose:**

The roles of blood low‐density lipoprotein cholesterol (LDL‐C), high‐density lipoprotein cholesterol (HDL‐C) and triglycerides in the development of post‐stroke dementia remain uncertain. This study was to investigate their potential associations.

**Methods:**

A retrospective cohort study was conducted using the Clinical Practice Research Datalink. Patients with first‐ever stroke but no prior dementia were followed up for 10 years. Cox regression was used to examine the association of baseline LDL‐C, HDL‐C and triglycerides with post‐stroke dementia.

**Results:**

Amongst 63,959 stroke patients, 15,879 had complete baseline data and were included in our main analysis. 10.8% developed dementia during a median of 4.6 years of follow‐up. The adjusted hazard ratio of dementia for LDL‐C (per log mmol/l increase) was 1.29 (95% confidence interval [CI] 1.14–1.47), with a linear increasing trend (*p* trend <0.001). The counterpart for triglycerides was 0.79 (95% CI 0.69–0.89), with a linear decreasing trend (*p* trend <0.001). For HDL‐C, there was no association with dementia (adjusted hazard ratio 0.89, 95% CI 0.74–1.08) or a linear trend (*p* trend = 0.22).

**Conclusions:**

Blood lipids may affect the risk of post‐stroke dementia in different ways, with higher risk associated with LDL‐C, lower risk associated with triglycerides, and no association with HDL‐C.

## Graphical abstract

Blood lipids may affect the risk of post‐stroke dementia in different ways, with higher risk associated with low‐density lipoprotein cholesterol, lower risk associated with triglycerides, and no association with high‐density lipoprotein cholesterol.

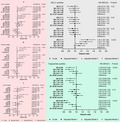

## BACKGROUND

The current American guideline recommends intensive statin therapy to reduce the blood low‐density lipoprotein cholesterol (LDL‐C) level following ischaemic stroke for prevention of future atherosclerotic cardiovascular disease but does not stipulate a target level of LDL‐C due to limited evidence [1]. European guidelines recommend a more intensive target of LDL‐C <1.4 mmol/l in very high risk patients compared with the previous target of <1.8 mmol/l [2, 3]. Whilst atherosclerotic cardiovascular disease and dementia share many cardiovascular risk factors [4], whether lipid levels affect the risk of dementia has not been established, with only low‐quality evidence available on the effect of lipid‐lowering treatment on the risk of dementia in the general population [5].

Relevant evidence is scarce in stroke patients, who are more likely to have hyperlipidaemia and develop subsequent dementia than the general population [6, 7]. According to a recent systematic review [8], only one randomised controlled trial, the Prevention of Decline in Cognition after Stroke Trial (PODCAST), has investigated the effect of lowering LDL‐C on post‐stroke dementia (PSD) [9]. This small‐scale trial found that an intensive target (LDL‐C <1.3 mmol/l vs. <3.0 mmol/l) significantly improved some cognitive test scores amongst 77 stroke patients during a 2‐year follow‐up, with a statistically non‐significant protective effect on PSD (odds ratio 0.18, 95% confidence interval [CI] 0.01–3.98). The same systematic review found no trials or observational studies for assessing the risk of PSD with different levels of high‐density lipoprotein cholesterol (HDL‐C) or triglycerides [8]. It is unclear whether target levels of LDL‐C recommended in the guidelines [2, 3], or different levels of other blood lipids, would have an impact on the occurrence of PSD.

This study was to examine the association of LDL‐C, HDL‐C and triglycerides with dementia in people with stroke.

## METHODS

### Data sources

A retrospective cohort study was conducted using the Clinical Practice Research Datalink (CPRD) GOLD with its linked data sources, including integrated Hospital Episode Statistics (HES), Office for National Statistics and Index of Multiple Deprivation [10]. The CPRD GOLD provides anonymised data extracted from primary care medical records, with coverage of a representative sample of approximately 7% of the UK population from more than 670 practices [10]. Read codes used in this study to define population, outcomes and comorbidities are publicly available on the website https://www.phpc.cam.ac.uk/pcu/research/research‐groups/crmh/cprd_cam/codelists/.

### Study population

**FIGURE 1:**
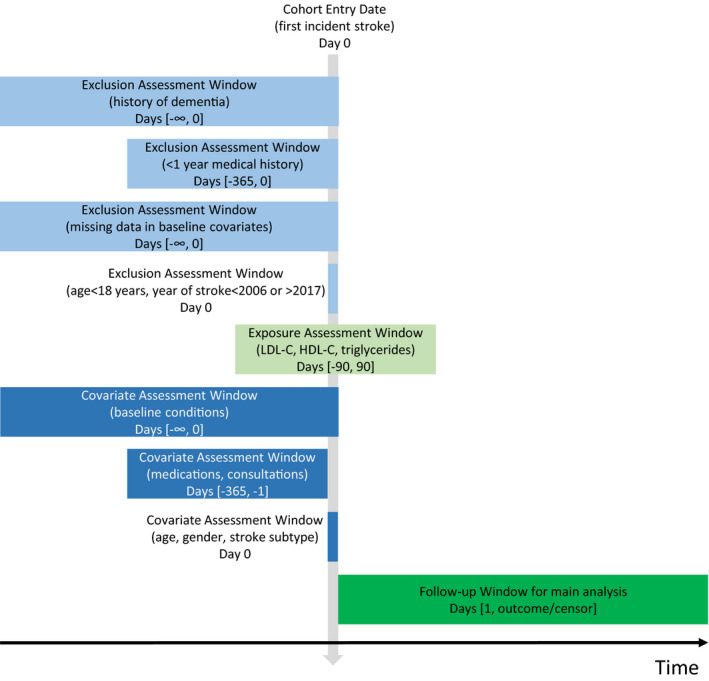
Study design diagram with measurement of exposures, outcomes and covariates. Abbreviations: HDL‐C, high‐density lipoprotein cholesterol; LDL‐C: low‐density lipoprotein cholesterol. [Color figure can be viewed at wileyonlinelibrary.com]

This study included patients aged at least 18 years with a diagnosis of first‐ever stroke (ischaemic stroke or intracerebral haemorrhage) recorded in the CPRD between 1 January 2006 and 31 December 2017. For those with stroke subtype unspecified in the CPRD, reference was made to HES via linkage to determine the subtype. In our main analysis, eligible patients were required to have complete baseline data for at least one lipid fraction. To ensure complete capture of pre‐existing diagnoses and medications, eligible patients were also required to have at least 12 months of record information before the index date of stroke. Patients with any dementia codes prior to the index stroke were excluded. The measurement of exposures, outcomes and covariates in relation to the timing of stroke is summarised in a study design diagram (Figure 1).

### Outcomes

The primary outcome of interest was incident PSD. A current consensus definition of PSD was adopted, which includes any subtype of dementia following stroke [11]. A clinical diagnosis of dementia was identified using Read codes recorded in the CPRD or International Classification of Diseases 10th version (ICD‐10) codes recorded in HES and the Office for National Statistics.

### Exposures

The exposure of interest was blood lipid fractions (i.e., LDL‐C, HDL‐C and triglycerides) at index stroke, which could help inform post‐stroke lipid target treatment. To account for the delay in post‐stroke lipid tests in clinical practice, the measures recorded within the 3 months before and after stroke were used (6 months centring around the index stroke) to represent the lipid level at baseline. When multiple measures were available, the average was used. Each lipid fraction was logarithmically transformed when treated as a continuous variable, and was further categorised into quintiles. For LDL‐C, it was also classified into ordinal categories reflecting the target levels recommended for lipid‐lowering treatment in the previous clinical guideline (1.8–2.59, 2.60–3.99, ≥4.0 vs. <1.8 mmol/l) and in the recent guideline with more intensive targets (1.4–1.79, 1.80–2.59, 2.6–2.99, ≥3.0 vs. <1.4 mmol/l) [2, 3].

### Potential confounders

Potential confounders included in this study represented demographics, lifestyle, cardiovascular factors, neuropsychological conditions, markers of immunity/inflammation, healthcare utilisation and medications, which are considered to be associated with, or may help reduce, the risk of dementia [5, 12](Appendix S1).

### Follow‐up

Follow‐up started from the index stroke until the occurrence of any dementia or censoring, whichever occurred earlier. Censoring included death, transfer‐out from the CPRD, the end of the 10‐year follow‐up or the last update of the CPRD (31 July 2018).

### Statistical analysis

Baseline characteristics were described and the difference between patients with and without blood lipids data was examined. The incidence rate of dementia was calculated. Crude and adjusted hazard ratios (HRs) with 95% CI of PSD over 10 years were estimated using Cox proportional hazards models for each lipid fraction as a continuous variable. Robust standard errors were used to allow for intragroup correlation related to possible clustering effects by general practice. Our primary model was conducted with full adjustment for demographics, lifestyle, the other two lipid fractions, comorbidity, healthcare utilisation and pre‐stroke medications. To explore how the association of interest was influenced by the potential confounders, two nested models were conducted with adjustment for some of these confounders (demographics, lifestyle, the other two lipid fractions and comorbidity; and demographics and lifestyle only). To accommodate their potential nonlinear relationships with dementia in all the models, body mass index (BMI) was logarithmically transformed and age and consultation were treated as restricted cubic smoothing spline variables with four knots [13]. The proportional hazards assumption was examined by testing the significance level of the interaction terms between each lipid fraction and time over 10 years. All models in the main analysis were complete‐case analyses.

The HR of dementia for each ordinal category of blood lipids (quintiles for each lipid fraction and different guideline targets for LDL‐C) was then estimated. The linear trend of the risk of dementia was tested by assigning median values of a lipid fraction to each ordinal category and treating them as a numerical variable in the models.

To examine robustness of the study results, a series of sensitivity analyses were conducted. (1) Multiple imputation was used with chained equations to replace missing baseline values (linear regression for the three lipid fractions and BMI [all in logarithmic form] and multinomial logistic regression for smoking status). Covariates included for the imputation consisted of an indicator variable for dementia and censoring, year of follow‐up and all independent variables involved in the primary model. As a rule of thumb, 75 imputations were conducted as there were 75% of patients having a missing value in at least one of the variables [14]. (2) Follow‐up was started at the fourth month post‐stroke. (3) Patients having a first record of dementia within the first 6 months after stroke were excluded. (4) Unspecified stroke was separated from ischaemic stroke. (5) The analysis was restricted to patients with linkage to HES data. (6) Competing‐risks regression models were used treating death as a competing‐risks event to calculate sub‐distribution HR.

Subgroup analysis by age group, gender, stroke subtype, cardiovascular comorbidity and pre‐stroke statin use was conducted using the primary model. Subgroup difference was examined by testing the interaction term between the stratifying variable and each lipid fraction.

To explore the potential impact of unmeasured confounding on our main findings, control outcome analysis was conducted using coronary heart disease (CHD) as a positive control outcome and fracture and peptic ulcer as negative control outcomes, which were selected based on the findings from large‐scale trials [15, 16, 17, 18, 19, 20, 21]. Whilst conducted for each lipid fraction, this analysis was primarily for LDL‐C given that the evidence on these outcomes for LDL‐C was more conclusive than that for HDL‐C and triglycerides. In this analysis, the patients who had the corresponding condition before stroke were additionally excluded from the analysis.

All data management and statistical analyses were conducted using Stata 15. Quality control was performed before analysis (Appendix S2). The statistical significance was *p* < 0.05 two‐tailed except for subgroup analysis, where a Bonferroni correction was applied to the significance level of subgroup difference that divided 0.05 by the 11 subgroups examined (i.e., 0.0045).

### Ethics approval and patient consents

Ethics approval was obtained from the Independent Scientific Advisory Committee of the CPRD (protocol number 17_201R), with no written consent from participants required.

## RESULTS

### Patient inclusion and baseline characteristics

Amongst 63,959 stroke patients, 17,729 patients had baseline values for LDL‐C, 22,436 for HDL‐C and 19,473 for triglycerides. 15,879 patients with complete baseline data contributed to the main modelling analysis (Appendix S3). In these patients, there was a higher proportion of younger people, males, ischaemic stroke, diabetes and prior treatments (statins and antidiabetic drugs) compared with those having missing lipid data (Table 1). Medians of LDL‐C, HDL‐C and triglycerides (mmol/l) were 2.4 (interquartile range 1.8–3.1), 1.3 (1.1–1.6) and 1.3 (1.0–1.8), respectively.

**TABLE 1:** Baseline characteristics of eligible patients

Characteristic (n, [%])	Total (*N* = 63,959)	Lipid data available			No lipid data (*n* = 40,065)	SMD^g^
		LDL cholesterol (*n* = 17,729)	HDL cholesterol (*n* = 22,436)	Triglycerides (*n* = 19,473)		
LDL mmol/l, median (IQR)^a^	NA	2.4 (1.8–3.1)	2.4 (1.8–3.1)	2.4 (1.8–3.1)	NA	NA
Log mmol/l^a^	NA	0.9 (0.6–1.1)	0.9 (0.6–1.1)	0.9 (0.6–1.1)	NA	NA
HDL mmol/l, median (IQR)^b^	NA	1.3 (1.1–1.6)	1.3 (1.1–1.6)	1.3 (1.1–1.6)	NA	NA
Log mmol/l^b^	NA	0.3 (0.1–0.5)	0.3 (0.1–0.5)	0.3 (0.1–0.5)	NA	NA
TG mmol/l, median (IQR)^c^	NA	1.3 (1.0–1.8)	1.3 (1.0–1.8)	1.3 (1.0–1.8)	NA	NA
Log mmol/l^c^	NA	0.3 (−0.1–0.6)	0.3 (0–0.6)	0.3 (0–0.6)	NA	NA
Age, years, median (IQR)	75 (64–83)	72 (63–80)	72 (63–80)	72 (63–80)	76 (66–84)	0.21
Female	31,457 (49.2)	8019 (45.2)	10,305 (45.9)	8896 (45.7)	20,460 (51.1)	0.10
IMD						0.08
Group 1 (least deprived)	13,398 (20.9)	4100 (23.1)	4914 (21.9)	4369 (22.4)	8120 (20.3)	
Group 2	11,951 (18.7)	3454 (19.5)	4405 (19.6)	3757 (19.3)	7310 (18.2)	
Group 3	13,778 (21.5)	3893 (22.0)	4947 (22.1)	4220 (21.7)	8538 (21.3)	
Group 4	13,023 (20.4)	3219 (18.2)	4181 (18.6)	3555 (18.3)	8578 (21.4)	
Group 5	11,809 (18.5)	3063 (17.3)	3989 (17.8)	3572 (18.3)	7519 (18.8)	
Smoking^d^						0.03
Current	12,625 (19.7)	3458 (19.5)	4419 (19.7)	3845 (19.7)	7887 (19.7)	
Former	20,775 (32.5)	5611 (31.7)	7157 (31.9)	6145 (31.6)	13,182 (32.9)	
Never	30,162 (47.2)	8594 (48.5)	10,771 (48.0)	9432 (48.4)	18,694 (46.7)	
BMI, median (IQR)^e^	26.6 (23.6–30.1)	26.9 (24.1–30.4)	27.0 (24.1–30.5)	27.0 (24.1–30.5)	26.4 (23.3–29.9)	0.13
Stroke subtype						0.15
Ischaemic stroke^f^	58,377 (91.3)	16,622 (93.8)	21,041 (93.8)	18,268 (93.8)	35,965 (89.8)	
Intracerebral haemorrhage	5582 (8.7)	1107 (6.2)	1395 (6.2)	1205 (6.2)	4100 (10.2)	
Atrial fibrillation	12,899 (20.2)	2928 (16.5)	3770 (16.8)	3212 (16.5)	8889 (22.2)	0.14
Alcohol problems	3039 (4.8)	764 (4.3)	998 (4.4)	843 (4.3)	1986 (5.0)	0.03
Anxiety	12,141 (19.0)	3385 (19.1)	4301 (19.2)	3734 (19.2)	7557 (18.9)	<0.01
Asthma	8253 (12.9)	2291 (12.9)	2919 (13.0)	2521 (12.9)	5147 (12.8)	<0.01
COPD	6210 (9.7)	1513 (8.5)	1954 (8.7)	1682 (8.6)	4114 (10.3)	0.05
Coronary heart disease	14,880 (23.3)	4077 (23.0)	5188 (23.1)	4487 (23.0)	9349 (23.3)	<0.01
Depression	16,974 (26.5)	4645 (26.2)	5954 (26.5)	5148 (26.4)	10,641 (26.6)	<0.01
Diabetes	11,429 (17.9)	3834 (21.6)	5008 (22.3)	4266 (21.9)	6142 (15.3)	0.17
Epilepsy	2020 (3.2)	468 (2.6)	620 (2.8)	513 (2.6)	1361 (3.4)	0.04
Hearing loss	13,994 (21.9)	3723 (21.0)	4730 (21.1)	4051 (20.8)	8976 (22.4)	0.03
Heart failure	5472 (8.6)	1138 (6.4)	1511 (6.7)	1260 (6.5)	3860 (9.6)	0.11
Hypertension	37,309 (58.3)	10,317 (58.2)	13,166 (58.7)	11,400 (58.5)	23,263 (58.1)	0.01
Parkinson's disease	828 (1.3)	163 (0.9)	206 (0.9)	178 (0.9)	610 (1.5)	0.06
Peripheral artery disease	3886 (6.1)	1028 (5.8)	1351 (6.0)	1131 (5.8)	2463 (6.1)	<0.01
Rheumatoid arthritis	4185 (6.5)	1015 (5.7)	1370 (6.1)	1127 (5.8)	2726 (6.8)	0.03
Transient ischaemic attack	6962 (10.9)	2183 (12.3)	2747 (12.2)	2361 (12.1)	4065 (10.1)	0.06
Consultation, median (IQR)	33 (20–50)	33 (20–49)	33 (20–49)	33 (20–49)	33 (20–50)	0.03
Statins	25,401 (39.7)	8092 (45.6)	10,269 (45.8)	8956 (46.0)	14,503 (36.2)	0.19
Other lipid‐lowering drugs	1868 (2.9)	667 (3.8)	834 (3.7)	750 (3.9)	989 (2.5)	0.07
Anticoagulant	4917 (7.7)	1202 (6.8)	1540 (6.9)	1323 (6.8)	3284 (8.2)	0.05
Antidiabetic drugs	8546 (13.4)	2886 (16.3)	3766 (16.8)	3208 (16.5)	4566 (11.4)	0.15
Antihypertensive drugs	40,926 (64.0)	11,327 (68.9)	14,515 (64.7)	12,530 (64.3)	25,461 (63.5)	0.02
Antiplatelets	25,435 (39.8)	7477 (42.2)	9522 (42.4)	8305 (42.6)	15,305 (38.2)	0.09

^a^ A total of 46,230 (72.3%) patients had a missing value of LDL cholesterol. Amongst those having a value of HDL cholesterol and triglycerides, 4889 (21.8%) and 2366 (12.2%) had a missing value of LDL cholesterol.

^b^ A total of 41,523 (64.9%) patients had a missing value of HDL cholesterol. Amongst those having a value of LDL cholesterol and triglycerides, 182 (1.0%) and 1307 (6.7%) had a missing value of HDL cholesterol.

^c^ A total of 44,486 (69.6%) patients had a missing value of triglycerides. Amongst those having a value of LDL cholesterol and HDL cholesterol, 622 (3.5%) and 4270 (19.0%) had a missing value of triglycerides.

^d^ A total of 397 (0.6%) patients had a missing value of smoking status: 66 (0.4%), 89 (0.4%), 51 (0.3%) and 302 (0.75%) for those with LDL cholesterol, HDL cholesterol, triglycerides and no lipid data, respectively.

^e^ A total of 5924 (9.3%) patients had a missing value of BMI: 1213 (6.8%), 1531 (6.8%), 1367 (7.0%) and 4260 (10.6%) for those with LDL cholesterol, HDL cholesterol, triglycerides and no lipid data, respectively.

^f^ A total of 28,000 (43.8%) patients had an unspecified stroke subtype: 8416 (47.5%), 10,532 (46.9%), 9316 (47.8%) and 16,761 (41.8%) for those with LDL cholesterol, HDL cholesterol, triglycerides and no lipid data, respectively.

^g^ SMD was used to examine the difference between those with and without available lipid data available. An absolute value of SMD larger than 0.10 indicated a significant difference.

Abbreviations: BMI, body mass index; COPD, chronic obstructive pulmonary disease; HDL, high‐density lipoprotein; IMD, Index of Multiple Deprivation; IQR, interquartile range; LDL, low‐density lipoprotein; NA, not applicable; SMD, standardised mean difference; TG, triglycerides.

Patients with higher LDL‐C were more likely to be younger and female, but less likely to have cardiovascular comorbidities, consult with their general practitioners or receive pre‐stroke medications (Appendix S4). Similar patterns were observed for HDL‐C except that patients in a higher‐level category tended to be older, female, less deprived, non‐smoker and have a lower BMI. For triglycerides, on the other hand, patients in a higher‐level category were more likely to be younger, more deprived and current smoker, and to have a higher BMI, CHD, diabetes, hypertension, more consultation and more pre‐stroke medications.

### Incidence rate of dementia

Amongst 15,879 patients with complete baseline data, 1713 (10.8%) patients developed PSD during a median of 4.6 years of follow‐up, with an incidence rate of 22.1 per 1000 person‐years. The incidence rate of PSD for each lipid fraction was similar (21.4, 22.1 and 21.1 per 1000 person‐years in patients with complete data on LDL‐C, HDL‐C and triglycerides, respectively) (Table 2). The rate increased with age regardless of gender (Appendix S5).

**TABLE 2:** Association of blood lipid fractions (per log mmol/l increase) with dementia

	LDL cholesterol	HDL cholesterol	Triglycerides
Total number	17,729	22,436	19,473
Cases with dementia	1860	2419	2065
Person‐years	86,716	109,428	97,863
Rate (per 1000 person‐years)	21.4	22.1	21.1
cHR (95% CI)^a^	0.89 (0.79–1.00)	1.46 (1.24–1.71)	0.65 (0.59–0.72)
aHR (95% CI) model 1^b^	1.08 (0.96–1.22)	0.92 (0.76–1.10)	0.91 (0.81–1.01)
Model 2^c^	1.26 (1.11–1.43)	0.90 (0.74–1.09)	0.80 (0.71–0.91)
Model 3^d^	1.29 (1.14–1.47)	0.89 (0.74–1.08)	0.79 (0.69–0.89)

15,879 stroke patients with complete baseline data were included in all the models for the HR estimates.

^a^ The HR was not adjusted for any potential confounders.

^b^ Model 1: adjusted for age (cubic spline variables), gender, IMD, smoking and BMI (logarithmic).

^c^ Model 2: adjusted for the variables in model 1 plus comorbidities (stroke subtype, atrial fibrillation, alcohol problem, anxiety, rheumatoid arthritis, asthma, chronic obstructive pulmonary disease, coronary heart disease, depression, diabetes, epilepsy, hearing loss, heart failure, hypertension, Parkinson's disease, peripheral artery disease and transient ischaemic attack) and the other two lipid fractions (log mmol/l).

^d^ Model 3: adjusted for the variables in model 2 plus consultation (cubic spline variables) and medications (statins, other lipid‐lowering drugs, anticoagulant, antiplatelet, antihypertensive drugs and antidiabetic drugs).

Abbreviations: aHR, adjusted hazard ratio; BMI, body mass index; cHR, crude hazard ratio; CI, confidence interval; HDL, high‐density lipoprotein; IMD, Index of Multiple Deprivation; LDL, low‐density lipoprotein.

### Association of LDL‐C with dementia

**FIGURE 2:**
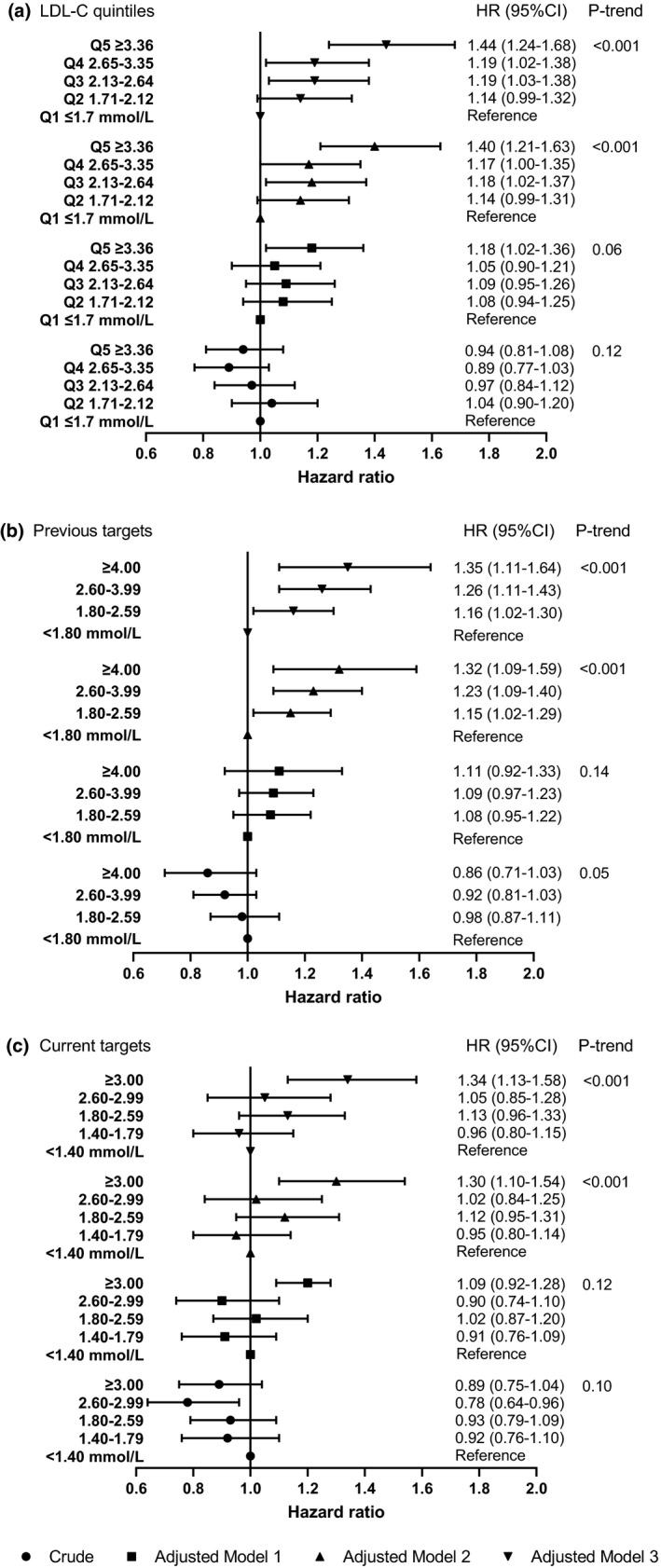
Association of LDL cholesterol levels with post‐stroke dementia. (a)–(c) reflect three criteria for LDL‐C classification (quintiles, previous and current guideline targets, respectively). 15,879 patients with complete baseline data were included in all the models. Tests for linear trend were conducted by assigning the medians of log LDL cholesterol to each level and treating the variable as a numerical variable in the Cox models. Crude: the HR was not adjusted for any potential confounders. Model 1: adjusted for age (cubic spline variables), gender, IMD, smoking and BMI (logarithmic). Model 2: adjusted for the variables in model 1 plus comorbidities (stroke subtype, atrial fibrillation, alcohol problem, anxiety, rheumatoid arthritis, asthma, chronic obstructive pulmonary disease, coronary heart disease, depression, diabetes, epilepsy, hearing loss, heart failure, hypertension, Parkinson's disease, peripheral artery disease and transient ischaemic attack) and the other two lipid fractions (log mmol/l). Model 3: adjusted for the variables in model 2 plus consultation (cubic spline variables) and medications (statins, other lipid‐lowering drugs, anticoagulant, antiplatelet, antihypertensive drugs and antidiabetic drugs). Abbreviations: BMI, body mass index; CI, confidence interval; HR, hazard ratio; IMD, Index of Multiple Deprivation; LDL‐C, low‐density lipoprotein cholesterol

Higher LDL‐C was associated with lower risk of PSD (crude HR 0.89, 95% CI 0.79–1.00) (Table 2). However, this association reversed in the adjusted estimates and became progressively stronger with adjustment for more potential confounders. The most important confounders were cardiovascular related comorbidities (Appendix S6). The fully adjusted HR was 1.29 (95% CI 1.14–1.47). Likewise, the fully adjusted estimates suggested that patients with a higher level of LDL‐C were more likely to develop PSD (Figure 2). The risk of dementia was significantly increased in a linear trend with an increase in LDL quintiles or guideline targets (all *p* values for trend <0.001). The proportional hazards assumption was met for all full adjustment models (all *p* values for interaction with time >0.05).

### Association of HDL‐C with dementia

**FIGURE 3:**
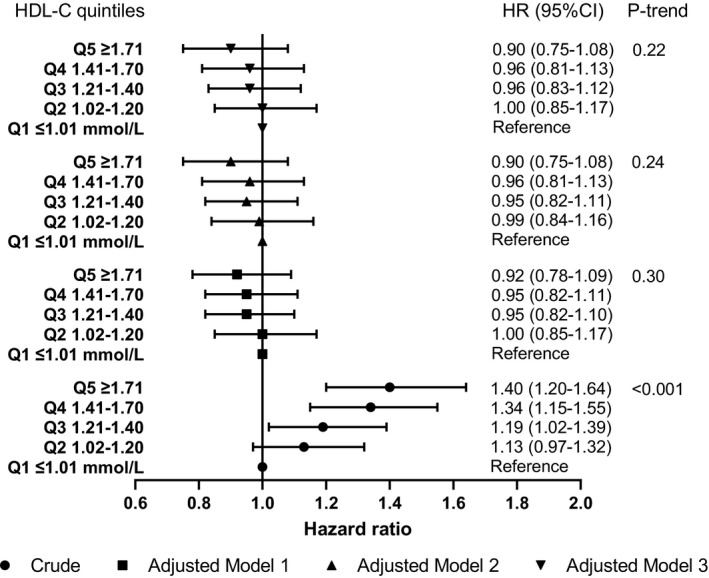
Association of HDL cholesterol levels with post‐stroke dementia. 15,879 patients with complete baseline data were included in all the models. Tests for linear trend were conducted by assigning the medians of log HDL cholesterol to each quintile and treating the variable as a numerical variable in the Cox models. Crude: the HR was not adjusted for any potential confounders. Model 1: adjusted for age (cubic spline variables), gender, IMD, smoking and BMI (logarithmic). Model 2: adjusted for the variables in model 1 plus comorbidities (stroke subtype, atrial fibrillation, alcohol problem, anxiety, rheumatoid arthritis, asthma, chronic obstructive pulmonary disease, coronary heart disease, depression, diabetes, epilepsy, hearing loss, heart failure, hypertension, Parkinson's disease, peripheral artery disease and transient ischaemic attack) and the other two lipid fractions (log mmol/l). Model 3: adjusted for the variables in model 2 plus consultation (cubic spline variables) and medications (statins, other lipid‐lowering drugs, anticoagulant, antiplatelet, antihypertensive drugs and antidiabetic drugs). Abbreviations: BMI, body mass index; CI, confidence interval; HDL‐C, high‐density lipoprotein cholesterol; HR, hazard ratio; IMD, Index of Multiple Deprivation

A higher level of HDL‐C was associated with higher risk of PSD (crude HR 1.46, 95% CI 1.24–1.71) (Table 2). However, this association became neutral in the adjusted estimates, with the fully adjusted HR being 0.89 (95% CI 0.74–1.08). When stratified into quintiles, no linear trend was observed (all *p* values for trend >0.05) after confounding adjustment despite a linear increasing trend in the crude estimate (*p* value for trend <0.001) (Figure 3). The proportional hazards assumption was met for all full adjustment models (all *p* values for interaction with time >0.05).

### Association of triglycerides with dementia

**FIGURE 4:**
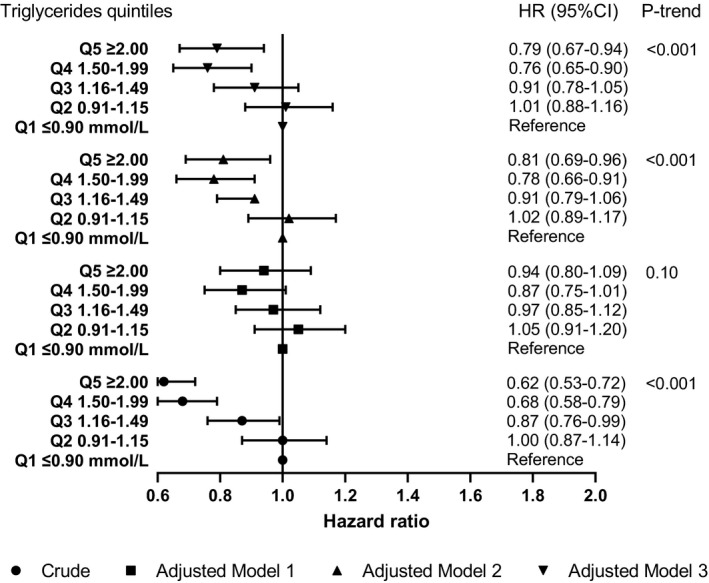
Association of triglyceride levels with post‐stroke dementia. 15,879 patients with complete baseline data were included in all the models. Tests for linear trend were conducted by assigning the medians of log triglycerides to each quintile and treating the variable as a numerical variable in the Cox models. Crude: the HR was not adjusted for any potential confounders. Model 1: adjusted for age (cubic spline variables), gender, IMD, smoking and BMI (logarithmic). Model 2: adjusted for the variables in model 1 plus comorbidities (stroke subtype, atrial fibrillation, alcohol problem, anxiety, rheumatoid arthritis, asthma, chronic obstructive pulmonary disease, coronary heart disease, depression, diabetes, epilepsy, hearing loss, heart failure, hypertension, Parkinson's disease, peripheral artery disease and transient ischaemic attack) and the other two lipid fractions (log mmol/l). Model 3: adjusted for the variables in model 2 plus consultation (cubic spline variables) and medications (statins, other lipid‐lowering drugs, anticoagulant, antiplatelet, antihypertensive drugs and antidiabetic drugs). Abbreviations: BMI, body mass index; CI, confidence interval; HR, hazard ratio; IMD, Index of Multiple Deprivation

Both crude and adjusted estimates suggested an inverse association of triglycerides with PSD (fully adjusted HR 0.79, 95% CI 0.69–0.89) (Table 2). The most important confounders were cardiovascular related comorbidities and other lipid fractions (Appendix S6). Similarly, a higher quintile level of triglycerides was associated with lower risk of PSD, with a linear decreasing trend (all *p* values for trend <0.001) (Figure 4). The proportional hazards assumption was met for all full adjustment models (all *p* values for interaction with time >0.05) except for the third quintile versus the lowest quintile (*p* value 0.03).

### Sensitivity analysis

Sensitivity analyses did not appreciably change the main results on the full adjusted estimates of the association of each lipid fraction with PSD (Appendix S7). After multiple imputation for missing data, a clearer linear trend was observed per level increase in LDL‐C and triglycerides than in the main analysis.

### Subgroup analysis

No evidence suggested that the association of LDL‐C, HDL‐C and triglycerides with PSD varied by stroke subtype and other baseline patient characteristics examined (Appendix S8). The increasing trends with a higher level of LDL‐C and the decreasing trends with a higher level of triglycerides persisted regardless of gender and hypertension. No significant linear trends were observed for HDL‐C in quintiles within any subgroups except for patients on prior statin treatment.

### Control outcome analysis

For CHD (positive control outcome), fully adjusted estimates showed an increased risk associated with LDL‐C, lower risk associated with HDL‐C and no association with triglycerides (Appendix S9). For the two negative control outcomes (fracture and peptic ulcer), no significant adjusted associations were observed for LDL‐C and triglycerides but a lower risk of fracture was associated with HDL‐C.

## DISCUSSION

### Summary of principal findings

In this study, LDL‐C was associated with a higher risk of PSD and there was a linear trend of increasing risk with a higher level of LDL‐C in quintiles and different guideline targets. Conversely, triglycerides were associated with a lower risk of PSD, with a linear decreasing trend for a higher level of triglycerides in quintiles. For HDL‐C, no association was observed in any adjusted estimates, with no clear linear trend. No evidence suggested that the association of LDL‐C, HDL‐C and triglycerides with PSD varied by stroke subtype and other baseline patient characteristics examined. For the control outcomes, LDL‐C was associated with CHD but not with fracture or peptic ulcer whilst no significant association was found for HDL‐C and triglycerides except that HDL‐C was inversely associated with CHD and positively with fracture.

### Strengths and limitations

Compared with previous studies identified in the recent systematic review [8], our study provided new evidence on the risk of PSD with different levels of blood lipid fractions, which may inform lipid target treatment following stroke. This study included a much larger sample of stroke patients, adjusted for more potential confounders, explored the impact of unmeasured confounding and reverse causality, and conducted a series of sensitivity analyses and subgroup analyses. The study also benefits from the strengths of the CPRD, such as representativeness of real practice settings, detailed prescription information, large sample size and long follow‐up [10].

However, there are limitations in this retrospective cohort study. First, only 25% of about 64,000 stroke patients in primary care had complete baseline data, with non‐primary‐care attenders not providing lipid information for our study. This could cause selection bias in our estimates and reduce generalisability to all stroke patients. However, multiple imputation did not change the conclusions from the complete‐case analysis but suggested a stronger linear trend for LDL‐C and triglycerides. Whilst some observed variables were found to predict missingness of blood lipids (Table 1), thus supporting our assumption of data ‘missing at random’, it remains possible that the multiple imputation may have introduced bias [14].

Second, some important potential risk factors of dementia could not be included for adjustment, such as severity and pathogenic mechanisms of stroke, size and location of cerebral lesion, education, ethnicity, physical activity and diet, which are poorly recorded in the CPRD. These factors, together with potential misclassification of baseline covariates, may have resulted in residual confounding. However, the impact of residual confounding may not be substantial for LDL‐C given that the association with control outcomes agreed with findings from trials.

Third, there is likely to be under‐recording of dementia in the CPRD due to the underdiagnosis issue in clinical practice [22]. Underdiagnosis of dementia could be more likely in patients with a higher level of LDL‐C or a lower level of triglycerides because they had less contact with health services (fewer prescriptions) due to fewer baseline comorbidities (Appendix S4) [23]. This means that the real association would in fact be stronger than our estimates for both lipid fractions if accounting for the underdiagnosis. Conversely, some evidence suggested an alternative scenario where people with more cardiovascular comorbidities tended to have underdiagnosis of dementia [24], in which case the real association could be weaker than our estimates. Which scenario was more likely in our study could not be determined, but the incidence of dementia observed was in line with meta‐analyses [7, 25] and the prospective cohort study [26] (Appendix S5). Our study was primarily focused on dementia, without considering its subtypes (the contemporary definition of PSD does not discriminate dementia subtypes [11]) and mild cognitive impairment, both of which were poorly recorded in the CPRD.

Fourth, our study only investigated baseline lipid levels and cannot provide information on time‐varying lipid levels after stroke, which may have affected our estimates of real association. Similarly, time‐varying confounders such as recurrent stroke and lipid‐lowering treatment over time were not considered.

Fifth, statistical power may not be sufficient to detect a significant difference in some of the trend and subgroup analyses.

Finally, reverse causality may exist in this study due to the measurement timeframe of blood lipid fractions and possible underlying cognitive impairment at baseline, but a series of analyses (control outcome analysis and sensitivity analysis) suggested that this was not an important determinant of our main results.

### Comparisons with other studies and possible mechanisms

As suggested by the recent systematic review [8], many studies have investigated the association of hypercholesterolaemia (defined by dichotomizing total cholesterol levels) with PSD or compared blood lipids (defined as continuous variables) between PSD and no PSD. However, their results were inconclusive due to some key limitations, including inadequate adjustment for confounders, selection bias, short‐term follow‐up, small sample size and selective reporting. Relevant evidence comparing the risk of PSD for different levels of lipid fractions is limited, with only one trial for LDL‐C [9] and no studies for HDL‐C or triglycerides. The small‐scale trial PODCAST observed a non‐significant protective effect of the intensive LDL‐C target on reducing the risk of PSD but some benefits for improving cognitive function related to vascular dementia such as executive function, response times and attention [9]. LDL‐C has been identified as a main factor for cerebral small vessel disease and non‐vascular neurodegenerative pathology as well as large vessel disease, which may contribute to the development of PSD [11, 27]. In line with these findings, our study suggested a positive association of LDL‐C with PSD.

Notably, our study found a linear decreasing trend in the risk of PSD with a higher level of triglycerides. The inverse association has also been reported in the general population in some studies [28, 29, 30, 31] but not in others [32, 33, 34, 35]. The studies [28, 29, 30, 31] observing the triglycerides paradox tended to include more older people and more participants within a guideline target of triglycerides (<1.7 mmol/l) [2], suggesting that the association may vary by age of triglycerides measurement and range of triglycerides. A low level of triglycerides may reflect poor nutrition [36], which could not be adjusted for in our study due to lack of data (except for BMI) in the CPRD. The triglycerides paradox was supported by the biological findings that triglycerides could protect cognition through increasing transport of peripheral ghrelin and insulin to the brain, increasing the expression of orexigenic hypothalamic peptides and decreasing the production of inflammatory cytokines and tissue responsiveness [37, 38, 39].

For CHD (positive control outcome), the inverse association with LDL‐C observed in our study agreed with the Treat Stroke to Target trial [15]. The neutral association between triglycerides and CHD was supported by the recent STRENGTH and OMEMI trials [40, 41] but opposite to the REDUCE‐IT trial [42]. For HDL‐C, large‐scale trials have failed to find cardiovascular benefits by increasing HDL level [43, 44, 45, 46]. The inverse association observed in our study and many other observational studies suggest that HDL‐C might just be a marker of cardiovascular health instead of a causal factor [47, 48].

For the negative control outcomes, no significant association was found except for the positive association between HDL‐C and fracture. Despite no relevant trial evidence, a recent Mendelian randomization study found a positive association for HDL‐C and no association for LDL‐C and triglycerides [49]. Meta‐analysis of observational studies also suggested that increased risk of fracture was associated with a higher level of HDL‐C but not LDL‐C or triglycerides [50].

### Implications for practice and future research

Intensive statin treatment to lower LDL‐C level in stroke patients may help to reduce the risk of dementia. It may be that guidelines should recommend lower LDL targets in people with stroke reflecting this benefit in addition to cardiovascular disease risk reduction.

Future trials to investigate the optimal target of LDL‐C in stroke patients should include cognitive outcomes to confirm our findings. These studies could focus on a more aggressive target of LDL‐C (e.g., <1.4 mmol/l), which might lead to further cognitive benefits as suggested in our study. More biological and population‐based studies are needed to confirm the inverse relationship between triglycerides and dementia.

## CONCLUSIONS

Blood lipids may affect the risk of PSD in different ways. Higher levels of LDL‐C are associated with increased risk of PSD, suggesting that a lower target of LDL‐C or intensive statin treatment may help reduce the risk of PSD. The association is unlikely to be substantially distorted by unmeasured confounding shared with CHD, fracture or peptic ulcer. Further trials are needed to confirm whether an aggressive target level of LDL‐C would be superior to a less aggressive target level in reducing the risk of PSD. More evidence is also needed to clarify the roles of triglycerides in the development of dementia.

## CONFLICT OF INTEREST

None.

## AUTHOR CONTRIBUTIONS

Zhirong Yang: Conceptualization (lead); data curation (lead); formal analysis (lead); funding acquisition (lead); investigation (lead); methodology (lead); resources (equal); software (lead); validation (lead); visualization (lead); writing—original draft (lead); writing—review and editing (lead). Duncan Edwards: Data curation (supporting); funding acquisition (supporting); investigation (supporting); project administration (supporting); resources (equal); validation (supporting); writing—review and editing (equal). Stephen Burgess: Formal analysis (supporting); resources (equal); supervision (supporting); validation (supporting); visualization (supporting); writing—review and editing (equal). Carol Brayne: Funding acquisition (supporting); investigation (supporting); resources (supporting); supervision (lead); validation (supporting); writing—review and editing (equal). Jonathan Mant: Conceptualization (supporting); funding acquisition (supporting); methodology (equal); project administration (lead); resources (equal); supervision (lead); validation (equal); visualization (equal); writing—review and editing (equal).

## Data Availability

The data that support the findings of this study are available from the CPRD but restrictions apply to the availability of these data, which were used under license for the current study and so are not publicly available.
