# Supplementary material for: Association of major blood lipids with post‐stroke dementia: A community‐based cohort study
Source: Eur J Neurol. 2022 Jan 26;29(4):968–79. doi: 10.1111/ene.15219 (PMC9303428; doi:10.1111/ene.15219)
Supplement: Supplementary file 1 [file ENE-29-968-s001.docx]

**Appendices**

**Association of major blood lipids with post-stroke dementia: a community-based cohort study**

Zhirong Yang ^1,2^, Duncan Edwards ^1^, Stephen Burgess ^3,4^, Carol Brayne ^5^, Jonathan Mant ^1^

^1^ Primary Care Unit, Department of Public Health and Primary Care, School of Clinical Medicine, University of Cambridge, Cambridge, UK

^2^ Shenzhen Institute of Advanced Technology, Chinese Academy of Sciences, Shenzhen, China

^3^ MRC Biostatistics Unit, School of Clinical Medicine, University of Cambridge, Cambridge, UK

^4^ Cardiovascular Epidemiology Unit, Department of Public Health and Primary Care, School of Clinical Medicine, University of Cambridge, Cambridge, UK

^5^ Cambridge Public Health, School of Clinical Medicine, University of Cambridge, Cambridge, UK

[Appendix S1. Potential confounders 1](#_Toc89715099)

[Appendix S2. Quality control criteria 3](#_Toc89715100)

[Appendix S3. Flow chart of patient inclusion 4](#_Toc89715101)

[Appendix S4. Baseline characteristics by level of each lipid fraction 5](#_Toc89715102)

[Appendix S5. Age and gender specific incidence rate of dementia 10](#_Toc89715103)

[Appendix S6. Alternative adjustment strategies for modelling 11](#_Toc89715104)

[Appendix S7. Sensitivity analyses 12](#_Toc89715105)

[Appendix S8. Subgroup analyses 18](#_Toc89715106)

[Appendix S9. Association of blood lipid fractions with control outcomes 21](#_Toc89715107)

# **Appendix S1. Potential confounders**

Potential confounders included in this study represented demographics, lifestyle, cardiovascular factors, neuropsychological conditions, markers of immunity/inflammation, health care utilisation and medications, which are considered to be associated with, or may help reduce, the risk of dementia. Demographic variables included age, gender and socioeconomic status. Age was calculated on the date of index stroke and categorised into four groups (18-64, 65-74, 75-84 and ≥85 years) for subgroup analysis. Index of Multiple Deprivation (IMD) grouped by quintile was used as an indicator of socioeconomic status. The IMD includes seven domains: income; employment; health and disability; education, skills and training; barriers to housing and services; crime; and living environment. Where patient-level IMD was missing, we used the general practice-level IMD. Smoking status was classified as current, former or never smoker. Body mass index (BMI) was analysed as a continuous variable. For both smoking and BMI variables, the most recent data before the index stroke were used to best represent their status at the date of index stroke. Stroke subtype was classified into ischaemic and haemorrhagic stroke (specific codes relating to ischaemic stroke or unspecified stroke codes were regarded as ischaemic stroke, considering these patients shared similar characteristics [1] and 90% of stroke in the UK is ischaemic stroke [2]). Prior comorbidity was defined as the presence of any relevant Read code or ICD-10 code before the index stroke. These conditions included atrial fibrillation, alcohol problem, anxiety, asthma, chronic obstructive pulmonary disease (COPD), depression, diabetes, epilepsy, heart failure, hearing loss, hypertension, Parkinson’s disease, rheumatoid arthritis, and transient ischaemic attack. The codes for each condition were developed as part of a project developing the Cambridge Multimorbidity Score [3] and are publicly available on the website: http://www.phpc.cam.ac.uk/pcu/cprd_cam/codelists/. We used the total number of consultations recorded within 365 days before index stroke as a measure of healthcare utilisation. Pre-stroke medications, including statins, other lipid-lowering drugs, anticoagulant, antiplatelet, antihypertensive drugs, and antidiabetic drugs, were defined using the CPRD product codes (equivalent of Gemscript codes) recorded during the 365 days prior to index stroke. For comorbidity and medication, an absence of related codes was regarded as an absence of the condition. There were other potential confounders not included in this study, such as ethnicity, education, genetics, physical activity and brain image. These variables are poorly recorded or unavailable in the data sources used.

References

1. Yang Z, Edwards D, Massou E, Saunders CL, Brayne C, Mant J. Statin use and high-dose statin use after ischemic stroke in the UK: a retrospective cohort study. *Clin Epidemiol*. 2019;11:495-508.

2. Feigin VL, Lawes CM, Bennett DA, Barker-Collo SL, Parag V. Worldwide stroke incidence and early case fatality reported in 56 population-based studies: a systematic review. *Lancet Neurol*. 2009;8(4):355-69.

3. Payne RA, Mendonca SC, Elliott MN, Saunders CL, Edwards DA, Marshall M, et al. Development and validation of the Cambridge Multimorbidity Score. *CMAJ*. 2020;192(5):E107-E14.

# **Appendix S2. Quality control criteria**

#

| **Data item** | **Unacceptable value** |
| --- | --- |
| **ALL the records of a patient were excluded for any reason below:** | |
| First registration date | Empty; invalid date; prior to year of birth; within one year before the first stroke diagnosis date |
| Current registration date | Invalid date; prior to first registration date; prior to year of birth |
| Transferred out date | Invalid date; present with no reason; prior to first registration date; prior to current registration date |
| A transfer-out reason | Present with no date |
| Registration status | Temporary patients |
| Age | Over 125 years at the end of follow-up |
| Year of birth | Absent |
| Gender | Other than male, female or indeterminate |
| Death date | Prior to the first registration date; prior to the current registration date |
| **RELEVANT episode records of a patient were excluded for any reason below:** | |
| Event date | Invalid; absent; prior to birth year |
| Weight | <30kg; >300kg |
| Height | <1.1 metres; >2.3 metres |
| LDL, HDL, triglycerides | >30mmol/L |
| **The date was CHANGED for any reason below:** | |
| Change the death date and transfer-out date to the first stroke diagnosis date | Death date prior to the first stroke diagnosis date; transfer-out date prior to the first stroke diagnosis date |
| Change the death date and transfer-out date to the first date of dementia diagnosis | Death date prior to the first date of dementia diagnosis; transfer-out date prior to the first date of dementia diagnosis |
| **The value of blood lipid was CHANGED to harmonise the measurement unit** | |
| Divide the value by 38.6 for LDL and HDL | Measured on mg per deciliter |
| Divide the value by 88.5 for triglycerides | Measured on mg per deciliter |

# **Appendix S3. Flow chart of patient inclusion**


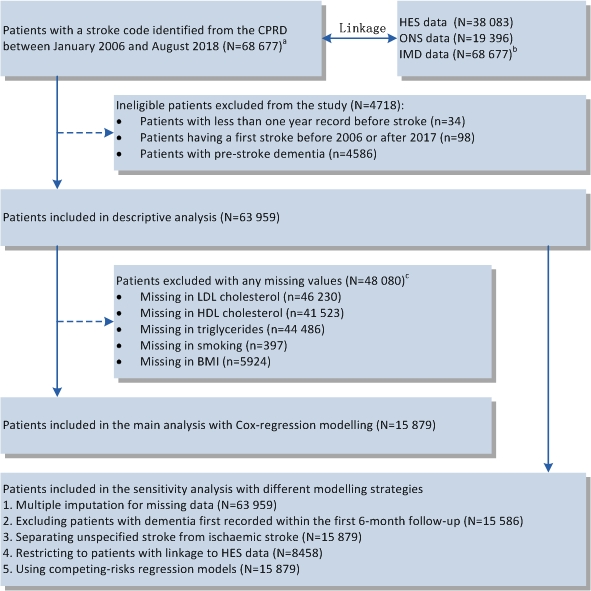


^d^

^c^

a. All the 68 677 patients identified in the CPRD were considered potentially eligible, regardless of whether they can be linked to other data sources.

b. In the IMD dataset, 38 616 patients had patient-level IMD and 30 061 patients had practice-level IMD.

c. To ensure there was at least 6-month follow-up for each patient (data used for this study were extracted from CPRD in August 2018), we excluded patients having a first stroke after December 2017.

d. Missingness in LDL cholesterol, HDL cholesterol, triglycerides, smoking and BMI was not mutually exclusive.

Abbreviations: BMI, body mass index; CPRD, Clinical Practice Research Datalink; HDL, high-density lipoprotein; HES, Hospital Episode Statistics; IMD, Index of Multiple Deprivation; LDL, low-density lipoprotein; ONS, Office for National Statistics.

# **Appendix S4. Baseline characteristics by level of each lipid fraction**

**Table S4-1. Baseline characteristics stratified by LDL cholesterol level (quintiles)**

| **Characteristic (n, [%])** | ≤ **1.7 mmol/L**  **(n=3925)** | **1.71-2.12**  **(n=3173)** | **2.13-2.64**  **(n=3547)** | **2.65-3.35**  **(n=3541)** | **≥3.36**  **(n=3543)** |
| --- | --- | --- | --- | --- | --- |
| Age, median (IQR) | 74 (65-81) | 73 (65-80) | 73 (64-79) | 71 (62-79) | 70 (60-78) |
| Female | 1561 (39.8) | 1350 (42.5) | 1537 (43.3) | 1670 (47.2) | 1901 (53.7) |
| IMD Group 1 (least deprived) | 884 (22.5) | 752 (23.7) | 830 (23.4) | 848 (24.0) | 786 (22.2) |
| Group 2 | 761 (19.4) | 609 (19.2) | 716 (20.2) | 672 (19.0) | 696 (19.6) |
| Group 3 | 821 (20.9) | 697 (22.0) | 792 (22.3) | 773 (21.8) | 810 (22.9) |
| Group 4 | 754 (19.2) | 574 (18.1) | 593 (16.7) | 627 (17.7) | 671 (18.9) |
| Group 5 | 705 (18.0) | 541 (17.0) | 616 (17.4) | 621 (17.5) | 580 (16.4) |
| Smoking Current ^a^ | 702 (17.9) | 574 (18.1) | 681 (19.2) | 756 (21.4) | 745 (21.0) |
| Former | 1312 (33.4) | 1060 (33.4) | 1166 (32.9) | 1067 (30.1) | 1006 (28.4) |
| Never | 1901 (48.4) | 1528 (48.2) | 1681 (47.4) | 1711 (48.3) | 1773 (50.0) |
| BMI, median (IQR) ^b^ | 27.2 (24.1-30.9) | 26.9 (24.1-30.6) | 27.0 (24.1-30.4) | 26.7 (23.9-30.1) | 26.8 (24.1-30.1) |
| Ischemic stroke ^c^ | 3715 (94.6) | 2997 (94.5) | 3342 (94.2) | 3286 (92.8) | 3282 (92.6) |
| Intracerebral hemorrhage | 210 (5.4) | 176 (5.5) | 205 (5.8) | 255 (7.2) | 261 (7.4) |
| Atrial fibrillation | 799 (20.4) | 615 (19.4) | 601 (16.9) | 518 (14.6) | 395 (11.1) |
| Alcohol problems | 156 (4.0) | 139 (4.4) | 149 (4.2) | 167 (4.7) | 153 (4.3) |
| Anxiety | 678 (17.3) | 570 (18.0) | 633 (17.8) | 700 (19.8) | 804 (22.7) |
| Asthma | 526 (13.4) | 389 (12.3) | 439 (12.4) | 450 (12.7) | 487 (13.7) |
| COPD | 394 (10.0) | 298 (9.4) | 271 (7.6) | 307 (8.7) | 243 (6.9) |
| Coronary heart disease | 1191 (30.3) | 885 (27.9) | 918 (25.9) | 595 (16.8) | 488 (13.8) |
| Depression | 961 (24.5) | 773 (24.4) | 895 (25.2) | 949 (26.8) | 1067 (30.1) |
| Diabetes | 1295 (33.0) | 769 (24.2) | 785 (22.1) | 546 (15.4) | 439 (12.4) |
| Epilepsy | 97 (2.5) | 73 (2.3) | 90 (2.5) | 102 (2.9) | 106 (3.0) |
| Hearing loss | 893 (22.8) | 708 (22.3) | 716 (20.2) | 746 (21.1) | 660 (18.6) |
| Heart failure | 376 (9.6) | 250 (7.9) | 209 (5.9) | 177 (5.0) | 126 (3.6) |
| Hypertension | 2478 (63.1) | 1988 (62.7) | 2104 (59.3) | 1950 (55.1) | 1797 (50.7) |
| Parkinson’s disease | 40 (1.0) | 23 (0.7) | 36 (1.0) | 35 (1.0) | 29 (0.8) |
| Peripheral artery disease | 271 (6.9) | 232 (7.3) | 215 (6.1) | 186 (5.3) | 124 (3.5) |
| Rheumatoid arthritis | 226 (5.8) | 179 (5.6) | 178 (5.0) | 199 (5.6) | 233 (6.6) |
| Transient ischemic attack | 496 (12.6) | 406 (12.8) | 453 (12.8) | 441 (12.5) | 387 (10.9) |
| Consultation, median (IQR) | 36 (22-53) | 34 (21-51) | 33 (20-48) | 30 (19-46) | 30 (17-44) |
| Statins | 2188 (55.7) | 1817 (57.3) | 1817 (51.2) | 1286 (36.3) | 984 (27.8) |
| Other lipid-lowering drugs | 133 (3.4) | 107 (3.4) | 141 (4.0) | 141 (4.0) | 145 (4.1) |
| Anticoagulant | 330 (8.4) | 251 (7.9) | 226 (6.4) | 216 (6.1) | 179 (5.1) |
| Antidiabetic drugs | 1042 (26.5) | 576 (18.2) | 569 (16.0) | 402 (11.4) | 297 (8.4) |
| Antihypertensive drugs | 2775 (70.7) | 2179 (68.7) | 2322 (65.5) | 2128 (60.1) | 1923 (54.3) |
| Antiplatelet | 1902 (48.5) | 1533 (48.3) | 1576 (44.4) | 1323 (37.4) | 1143 (32.3) |

a. A total of 66 (0.4%) patients had missing value of smoking status: 10 (0.3%), 11 (0.4%), 19 (0.5%), 7 (0.2%), and 19 (0.5%) for each quintile, respectively.

b. A total of 1213 (6.8%) patients had missing value of BMI: 280 (7.1%), 190 (6.0%), 222 (6.3%), 223 (6.3%), and 298 (8.4%) for each quintile, respectively.

c. A total of 8416 (47.5%) patients had an unspecified stroke subtype: 1824 (46.5%), 1453 (45.8%), 1631 (46.0%), 1734 (49.0%), and 1774 (50.0%) for each quintile, respectively.

Abbreviations: BMI, body mass index; COPD, chronic obstructive pulmonary disease; IMD, Index of Multiple Deprivation; IQR, interquartile range.

**Table S4-2. Baseline characteristics stratified by LDL cholesterol level (previous guideline targets)**

| **Characteristic (n, [%])** | **<1.8 mmol/L**  **(n=4667)** | **1.8-2.59**  **(n=5909)** | **2.60-3.99**  **(n=5517)** | **≥4.0**  **(n=1636)** |
| --- | --- | --- | --- | --- |
| Age, median (IQR) | 74 (65-81) | 73 (64-80) | 71 (62-79) | 69 (60-78) |
| Female | 1860 (39.9) | 2557 (43.3) | 2654 (48.1) | 948 (57.9) |
| IMD Group 1 (least deprived) | 1062 (22.8) | 1387 (23.5) | 1274 (23.1) | 377 (23.0) |
| Group 2 | 900 (19.3) | 1171 (19.8) | 1069 (19.3) | 314 (19.2) |
| Group 3 | 976 (20.9) | 1317 (22.3) | 1209 (21.9) | 391 (23.9) |
| Group 4 | 899 (19.3) | 1015 (17.2) | 1009 (18.3) | 296 (18.1) |
| Group 5 | 830 (17.8) | 1019 (17.2) | 956 (17.3) | 258 (15.8) |
| Smoking Current ^a^ | 837 (17.9) | 1104 (18.7) | 1144 (20.7) | 373 (22.8) |
| Former | 1572 (33.7) | 1947 (32.9) | 1649 (29.9) | 443 (27.1) |
| Never | 2245 (48.1) | 2831 (47.9) | 2710 (49.1) | 808 (49.4) |
| BMI, median (IQR) ^b^ | 27.2 (24.2-30.9) | 26.9 (24.1-30.4) | 26.8 (23.9-30.0) | 26.8 (24.2-30.3) |
| Ischemic stroke ^c^ | 4424 (94.8) | 5562 (94.1) | 5125 (92.9) | 1511 (92.4) |
| Intracerebral hemorrhage | 243 (5.2) | 347 (5.9) | 392 (7.1) | 125 (7.6) |
| Atrial fibrillation | 958 (20.5) | 1047 (17.7) | 758 (13.7) | 165 (10.1) |
| Alcohol problems | 185 (4.0) | 258 (4.4) | 244 (4.4) | 77 (4.7) |
| Anxiety | 814 (17.4) | 1050 (17.8) | 1127 (20.4) | 394 (24.1) |
| Asthma | 635 (13.6) | 716 (12.1) | 721 (13.1) | 219 (13.4) |
| COPD | 461 (9.9) | 497 (8.4) | 440 (8.0) | 115 (7.0) |
| Coronary heart disease | 1422 (30.5) | 1550 (26.2) | 887 (16.1) | 218 (13.3) |
| Depression | 1135 (24.3) | 1473 (24.9) | 1521 (27.6) | 516 (31.5) |
| Diabetes | 1491 (31.9) | 1344 (22.7) | 811 (14.7) | 188 (11.5) |
| Epilepsy | 114 (2.4) | 145 (2.5) | 157 (2.8) | 52 (3.2) |
| Hearing loss | 1068 (22.9) | 1239 (21.0) | 1121 (20.3) | 295 (18.0) |
| Heart failure | 446 (9.6) | 384 (6.5) | 258 (4.7) | 50 (3.1) |
| Hypertension | 2962 (63.5) | 3562 (60.3) | 2969 (53.8) | 824 (50.4) |
| Parkinson’s disease | 48 (1.0) | 50 (0.8) | 52 (0.9) | 13 (0.8) |
| Peripheral artery disease | 328 (7.0) | 390 (6.6) | 252 (4.6) | 58 (3.5) |
| Rheumatoid arthritis | 274 (5.9) | 308 (5.2) | 333 (6.0) | 100 (6.1) |
| Transient ischemic attack | 588 (12.6) | 762 (12.9) | 653 (11.8) | 180 (11.0) |
| Consultation, median (IQR) | 36 (22-53) | 33 (20-49) | 30 (18-46) | 30 (17-44) |
| Statins | 2625 (56.3) | 3163 (53.5) | 1843 (33.4) | 461 (28.2) |
| Other lipid-lowering drugs | 153 (3.3) | 227 (3.8) | 219 (4.0) | 68 (4.2) |
| Anticoagulant | 393 (8.4) | 412 (7.0) | 311 (5.6) | 86 (5.3) |
| Antidiabetic drugs | 1183 (25.3) | 993 (16.8) | 574 (10.4) | 136 (8.3) |
| Antihypertensive drugs | 3300 (70.7) | 3923 (66.4) | 3238 (58.7) | 866 (50.9) |
| Antiplatelet | 2276 (48.8) | 2707 (45.8) | 1999 (36.2) | 495 (30.3) |

a. A total of 66 (0.4%) patients had missing value of smoking status: 13 (0.3%), 27 (0.5%), 14 (0.3%), and 12 (0.7%) for each target group, respectively.

b. A total of 1213 (6.8%) patients had missing value of BMI: 319 (6.8%), 365 (6.2%), 378 (6.9%), and 151 (9.2%) for each target group, respectively.

c. A total of 8416 (47.5%) patients had an unspecified stroke subtype: 2161 (46.3%), 2719 (46.0%), 2718 (49.3%), and 818 (50.0%) for each target group, respectively.

Abbreviations: BMI, body mass index; COPD, chronic obstructive pulmonary disease; IMD, Index of Multiple Deprivation; IQR, interquartile range.

**Table S4-3. Baseline characteristics stratified by LDL cholesterol level (current guideline targets)**

| **Characteristic (n, [%])** | **<1.4 mmol/L**  **(n=1925)** | **1.4-1.79**  **(n=2742)** | **1.80-2.59**  **(n=5909)** | **2.6-2.99**  **(n=1863)** | **≥3.0**  **(n=5290)** |
| --- | --- | --- | --- | --- | --- |
| Age, median (IQR) | 74 (65-81) | 74 (65-81) | 73 (64-80) | 71 (62-79) | 70 (60-78) |
| Female | 759 (39.4) | 1101 (40.2) | 2557 (43.3) | 842 (45.2) | 2760 (52.2) |
| IMD Group 1 (least deprived) | 416 (21.6) | 646 (23.6) | 1387 (23.5) | 450 (24.2) | 1201 (22.7) |
| Group 2 | 367 (19.1) | 533 (19.4) | 1171 (19.8) | 347 (18.6) | 1036 (19.6) |
| Group 3 | 398 (20.7) | 578 (21.1) | 1317 (22.3) | 412 (22.1) | 1188 (22.5) |
| Group 4 | 377 (19.6) | 522 (19.0) | 1015 (17.2) | 328 (17.6) | 977 (18.5) |
| Group 5 | 367 (19.1) | 463 (16.9) | 1019 (17.2) | 326 (17.5) | 888 (16.8) |
| Smoking Current ^a^ | 339 (17.6) | 498 (18.2) | 1104 (18.7) | 398 (21.4) | 1119 (21.2) |
| Former | 649 (33.7) | 923 (33.7) | 1947 (32.9) | 566 (30.4) | 1526 (28.8) |
| Never | 934 (48.5) | 1311 (47.8) | 2831 (47.9) | 898 (48.2) | 2620 (49.5) |
| BMI, median (IQR) ^b^ | 27.6  (24.3-31.3) | 27.0  (24.1-30.5) | 26.9  (24.1-30.4) | 26.7  (23.7-30.0) | 26.8  (24.1-30.1) |
| Ischemic stroke ^c^ | 1824 (94.8) | 2600 (94.8) | 5562 (94.1) | 1729 (92.8) | 4907 (92.8) |
| Intracerebral hemorrhage | 101 (5.2) | 142 (5.2) | 347 (5.9) | 134 (7.2) | 383 (7.2) |
| Atrial fibrillation | 392 (20.4) | 566 (20.6) | 1047 (17.7) | 289 (15.5) | 634 (12.0) |
| Alcohol problems | 81 (4.2) | 104 (3.8) | 258 (4.4) | 85 (4.6) | 236 (4.5) |
| Anxiety | 340 (17.7) | 474 (17.3) | 1050 (17.8) | 379 (20.3) | 1142 (21.6) |
| Asthma | 275 (14.3) | 360 (13.1) | 716 (12.1) | 235 (12.6) | 705 (13.3) |
| COPD | 222 (11.5) | 239 (8.7) | 497 (8.4) | 172 (9.2) | 383 (7.2) |
| Coronary heart disease | 573 (29.8) | 849 (31.0) | 1550 (26.2) | 357 (19.2) | 748 (14.1) |
| Depression | 463 (24.1) | 672 (24.5) | 1473 (24.9) | 492 (26.4) | 1545 (29.2) |
| Diabetes | 716 (37.2) | 775 (28.3) | 1344 (22.7) | 292 (15.7) | 707 (13.4) |
| Epilepsy | 51 (2.6) | 63 (2.3) | 145 (2.5) | 45 (2.4) | 164 (3.1) |
| Hearing loss | 448 (23.3) | 620 (22.6) | 1239 (21.0) | 402 (21.6) | 1014 (19.2) |
| Heart failure | 190 (9.9) | 256 (9.3) | 384 (6.5) | 108 (5.8) | 200 (3.8) |
| Hypertension | 1229 (63.8) | 1733 (63.2) | 3562 (60.3) | 1049 (56.3) | 2744 (51.9) |
| Parkinson’s disease | 19 (1.0) | 29 (1.1) | 50 (0.8) | 26 (1.4) | 39 (0.7) |
| Peripheral artery disease | 120 (6.2) | 208 (7.6) | 390 (6.6) | 96 (5.2) | 214 (4.0) |
| Rheumatoid arthritis | 119 (6.2) | 155 (5.7) | 308 (5.2) | 103 (5.5) | 330 (6.2) |
| Transient ischemic attack | 237 (12.3) | 351 (12.8) | 762 (12.9) | 238 (12.8) | 595 (11.2) |
| Consultation, median (IQR) | 36 (23-54) | 35 (21-52) | 33 (20-49) | 31 (19-46) | 30 (18-45) |
| Statins | 1076 (55.9) | 1549 (56.5) | 3163 (53.5) | 736 (39.5) | 1568 (29.6) |
| Other lipid-lowering drugs | 66 (3.4) | 87 (3.2) | 227 (3.8) | 70 (3.8) | 217 (4.1) |
| Anticoagulant | 174 (9.0) | 219 (8.0) | 412 (7.0) | 114 (6.1) | 283 (5.3) |
| Antidiabetic drugs | 593 (30.8) | 590 (21.5) | 993 (16.8) | 219 (11.8) | 491 (9.3) |
| Antihypertensive drugs | 1377 (71.5) | 1923 (70.1) | 3923 (66.4) | 1131 (60.7) | 2973 (56.2) |
| Antiplatelet | 927 (48.2) | 1349 (49.2) | 2707 (45.8) | 722 (38.8) | 1772 (33.5) |

a. A total of 66 (0.4%) patients had missing value of smoking status: 3 (0.2%), 10 (0.4%), 27 (0.5%), 1 (0.1%), and 25 (0.5%) for each target group, respectively.

b. A total of 1213 (6.8%) patients had missing value of BMI: 123 (6.4%), 196 (7.2%), 365 (6.2%), 126 (6.8%), and 403 (7.6%) for each target group, respectively.

c. A total of 8416 (47.5%) patients had an unspecified stroke subtype: 912 (47.4%), 1249 (45.6%), 2719 (46.0%), 885 (47.5%), and 2651 (50.1%) for each target group, respectively.

Abbreviations: BMI, body mass index; COPD, chronic obstructive pulmonary disease; IMD, Index of Multiple Deprivation; IQR, interquartile range.

**Table S4-4. Baseline characteristics stratified by HDL cholesterol level (quintiles)**

| **Characteristic (n, [%])** | ≤ **1.01 mmol/L**  **(n=4554)** | **1.02-1.20**  **(n=4557)** | **1.21-1.40**  **(n=4352)** | **1.41-1.70**  **(n=4607)** | **≥1.71**  **(n=4366)** |
| --- | --- | --- | --- | --- | --- |
| Age, median (IQR) | 69 (60-77) | 71 (62-79) | 73 (64-80) | 74 (65-81) | 75 (66-82) |
| Female | 1074 (23.6) | 1539 (33.8) | 2005 (46.1) | 2639 (57.3) | 3048 (69.8) |
| IMD Group 1 (least deprived) | 906 (19.9) | 928 (20.4) | 951 (21.8) | 1062 (23.0) | 1067 (24.4) |
| Group 2 | 839 (18.4) | 892 (19.6) | 839 (19.3) | 920 (20.0) | 915 (21.0) |
| Group 3 | 987 (21.7) | 990 (21.7) | 981 (22.5) | 1046 (22.7) | 943 (21.6) |
| Group 4 | 915 (20.1) | 871 (19.1) | 821 (18.9) | 848 (18.4) | 726 (16.6) |
| Group 5 | 907 (19.9) | 876 (19.2) | 760 (17.5) | 721 (15.9) | 715 (16.4) |
| Smoking Current ^a^ | 1103 (24.2) | 947 (20.8) | 821 (18.9) | 797 (17.3) | 751 (17.2) |
| Former | 1591 (34.9) | 1496 (32.8) | 1422 (32.7) | 1427 (31.0) | 1221 (28.0) |
| Never | 1834 (40.3) | 2092 (45.9) | 2094 (48.1) | 2368 (51.4) | 2383 (54.6) |
| BMI, median (IQR) ^b^ | 28.9 (26.0-32.6) | 27.8 (25.0-31.2) | 27.2 (24.5-30.5) | 26.2 (23.4-29.5) | 24.9 (22.2-27.8) |
| Ischemic stroke ^c^ | 4271 (93.8) | 4294 (94.2) | 4104 (94.3) | 4325 (93.9) | 4047 (92.7) |
| Intracerebral hemorrhage | 283 (6.2) | 263 (5.8) | 248 (5.7) | 282 (6.1) | 319 (7.3) |
| Atrial fibrillation | 816 (17.9) | 788 (17.3) | 693 (15.9) | 778 (16.9) | 695 (15.9) |
| Alcohol problems | 232 (5.1) | 201 (4.4) | 172 (4.0) | 173 (3.8) | 220 (5.0) |
| Anxiety | 823 (18.1) | 823 (18.1) | 805 (18.5) | 912 (19.8) | 938 (21.5) |
| Asthma | 550 (12.1) | 551 (12.1) | 545 (12.5) | 632 (13.7) | 641 (14.7) |
| COPD | 398 (8.7) | 361 (7.9) | 322 (7.4) | 380 (8.2) | 493 (11.3) |
| Coronary heart disease | 1441 (31.6) | 1124 (24.7) | 981 (22.5) | 918 (19.9) | 724 (16.6) |
| Depression | 1179 (25.9) | 1187 (26.0) | 1145 (26.3) | 1263 (47.2) | 1180 (27.0) |
| Diabetes | 1659 (36.4) | 1186 (26.0) | 919 (21.1) | 749 (16.3) | 495 (11.3) |
| Epilepsy | 113 (2.5) | 108 (2.4) | 98 (2.3) | 136 (3.0) | 165 (3.8) |
| Hearing loss | 908 (19.9) | 915 (20.1) | 884 (20.3) | 991 (21.5) | 1032 (23.6) |
| Heart failure | 440 (9.7) | 323 (7.1) | 262 (6.0) | 262 (5.7) | 224 (5.1) |
| Hypertension | 2717 (59.7) | 2683 (58.9) | 2579 (59.3) | 2688 (58.3) | 2499 (57.2) |
| Parkinson’s disease | 31 (0.7) | 39 (0.9) | 41 (0.9) | 45 (1.0) | 50 (1.1) |
| Peripheral artery disease | 379 (8.3) | 284 (6.2) | 257 (5.9) | 235 (5.1) | 196 (4.5) |
| Rheumatoid arthritis | 218 (4.8) | 245 (5.4) | 264 (6.1) | 303 (6.6) | 340 (7.8) |
| Transient ischemic attack | 547 (12.0) | 545 (12.0) | 507 (11.6) | 565 (12.3) | 583 (13.4) |
| Consultation, median (IQR) | 35 (21-52) | 32 (19-49) | 32 (20-47) | 33 (20-49) | 33 (21-48) |
| Statins | 2463 (54.1) | 2198 (48.2) | 1980 (45.5) | 1964 (42.6) | 1664 (38.1) |
| Other lipid-lowering drugs | 241 (5.3) | 175 (3.8) | 138 (3.2) | 151 (3.3) | 129 (3.0) |
| Anticoagulant | 395 (8.7) | 335 (7.4) | 281 (6.5) | 285 (6.2) | 244 (5.6) |
| Antidiabetic drugs | 1318 (28.9) | 913 (20.0) | 674 (15.5) | 520 (11.3) | 341 (7.8) |
| Antihypertensive drugs | 3096 (68.0) | 2946 (64.6) | 2811 (64.6) | 2954 (64.1) | 2708 (62.0) |
| Antiplatelet | 2132 (46.8) | 1990 (43.7) | 1784 (41.0) | 1878 (40.8) | 1738 (39.8) |

a. A total of 89 (0.4%) patients had missing value of smoking status: 26 (0.6%), 22 (0.5%), 15 (0.3%), 15 (0.3%), and 11 (0.3%) for each quintile, respectively.

b. A total of 1519 (6.8%) patients had missing value of BMI: 280 (6.2%), 303 (6.7%), 317 (7.3%), 292 (6.3%), and 327 (7.5%) for each quintile, respectively.

c. A total of 10 532 (46.9%) patients had an unspecified stroke subtype: 1982 (43.5%), 2117 (46.5%), 2059 (47.3%), 2213 (48.0%), and 2161 (49.5%) for each quintile, respectively.

Abbreviations: BMI, body mass index; COPD, chronic obstructive pulmonary disease; IMD, Index of Multiple Deprivation; IQR, interquartile range.

**Table S4-5. Baseline characteristics stratified by triglycerides level (quintiles)**

| **Characteristic (n, [%])** | ≤ **0.9 mmol/L**  **(n=4444)** | **0.91-1.15**  **(n=3355)** | **1.16-1.49**  **(n=3903)** | **1.50-1.99**  **(n=3881)** | **≥ 2.0**  **(n=3890)** |
| --- | --- | --- | --- | --- | --- |
| Age, median (IQR) | 74 (66-81) | 74 (65-81) | 73 (65-80) | 71 (62-78) | 68 (59-76) |
| Female | 1913 (43.0) | 1555 (46.3) | 1942 (49.8) | 1818 (46.8) | 1668 (42.9) |
| IMD Group 1 (least deprived) | 1130 (25.4) | 777 (23.2) | 897 (23.0) | 843 (21.7) | 722 (18.6) |
| Group 2 | 922 (20.7) | 658 (19.6) | 736 (18.9) | 755 (19.5) | 686 (17.6) |
| Group 3 | 950 (21.4) | 713 (21.3) | 896 (23.0) | 809 (20.8) | 852 (21.9) |
| Group 4 | 770 (17.3) | 625 (18.6) | 661 (16.9) | 722 (18.6) | 777 (20.0) |
| Group 5 | 672 (15.1) | 582 (17.3) | 713 (18.3) | 752 (19.4) | 853 (21.9) |
| Smoking Current ^a^ | 674 (15.2) | 573 (17.1) | 748 (19.2) | 828 (21.3) | 1022 (26.3) |
| Former | 1385 (31.2) | 1041 (31.0) | 1236 (31.7) | 1226 (31.6) | 1257 (32.3) |
| Never | 2376 (53.5) | 1732 (51.6) | 1907 (48.9) | 1815 (46.8) | 1602 (41.2) |
| BMI, median (IQR) ^b^ | 25.2 (22.8-28.1) | 26.2 (23.4-29.5) | 27.0 (24.2-30.5) | 28.0 (25.1-31.3) | 28.7 (25.9-32.3) |
| Ischemic stroke ^c^ | 4160 (93.6) | 3169 (94.5) | 3660 (93.8) | 3623 (93.4) | 3656 (94.0) |
| Intracerebral hemorrhage | 284 (6.4) | 186 (5.5) | 243 (6.2) | 258 (6.6) | 234 (6.0) |
| Atrial fibrillation | 869 (19.6) | 610 (18.2) | 679 (17.4) | 564 (14.5) | 490 (12.6) |
| Alcohol problems | 179 (4.0) | 133 (4.0) | 168 (4.3) | 178 (4.6) | 185 (4.8) |
| Anxiety | 740 (16.7) | 628 (18.7) | 707 (18.1) | 800 (20.6) | 859 (22.1) |
| Asthma | 552 (12.4) | 403 (12.0) | 518 (13.3) | 514 (13.2) | 534 (13.7) |
| COPD | 379 (8.5) | 272 (8.1) | 355 (9.1) | 315 (8.1) | 361 (9.3) |
| Coronary heart disease | 962 (21.6) | 779 (23.2) | 882 (22.6) | 879 (22.7) | 985 (25.3) |
| Depression | 971 (21.8) | 794 (23.7) | 1018 (26.1) | 1103 (28.4) | 1262 (32.4) |
| Diabetes | 627 (14.1) | 608 (18.1) | 787 (20.2) | 964 (24.8) | 1280 (32.9) |
| Epilepsy | 103 (2.3) | 93 (2.8) | 110 (2.8) | 106 (2.7) | 101 (2.6) |
| Hearing loss | 1057 (23.8) | 736 (21.9) | 775 (19.9) | 791 (20.4) | 692 (17.8) |
| Heart failure | 286 (6.4) | 218 (6.5) | 250 (6.4) | 244 (6.3) | 262 (6.7) |
| Hypertension | 2506 (56.4) | 1924 (57.3) | 2303 (59.0) | 2320 (59.8) | 2347 (60.3) |
| Parkinson’s disease | 68 (1.5) | 29 (0.9) | 29 (0.7) | 27 (0.7) | 25 (0.6) |
| Peripheral artery disease | 221 (5.0) | 166 (5.0) | 239 (6.1) | 237 (6.1) | 268 (6.9) |
| Rheumatoid arthritis | 231 (5.2) | 175 (5.2) | 241 (6.2) | 239 (6.2) | 241 (6.2) |
| Transient ischemic attack | 554 (12.5) | 409 (12.2) | 470 (12.0) | 467 (12.0) | 461 (11.9) |
| Consultation, median (IQR) | 31 (19-46) | 32 (19-48) | 32 (20-48) | 33 (20-49) | 36 (21-53) |
| Statins | 1726 (38.8) | 1480 (44.1) | 1735 (44.5) | 1915 (49.3) | 2100 (54.0) |
| Other lipid-lowering drugs | 118 (2.7) | 90 (2.7) | 126 (3.2) | 168 (4.3) | 248 (6.4) |
| Anticoagulant | 302 (6.8) | 238 (7.1) | 270 (6.9) | 236 (6.1) | 277 (7.1) |
| Antidiabetic drugs | 448 (10.1) | 440 (13.1) | 579 (14.8) | 752 (19.4) | 989 (25.4) |
| Antihypertensive drugs | 2727 (61.4) | 2118 (63.1) | 2507 (64.2) | 2562 (66.0) | 2616 (67.2) |
| Antiplatelet | 1857 (41.8) | 1445 (43.1) | 1650 (42.3) | 1647 (42.4) | 1706 (43.9) |

a. A total of 51 (0.3%) patients had missing value of smoking status: 9 (0.2%), 9 (0.3%), 12 (0.3%), 12 (0.3%), and 9 (0.2%) for each quintile, respectively.

b. A total of 1367 (7.0%) patients had missing value of BMI: 334 (7.5%), 233 (6.9%), 265 (6.8%), 264 (6.8%), and 271 (7.0%) for each quintile, respectively.

c. A total of 9316 (47.8%) patients had an unspecified stroke subtype: 2067 (46.5%), 1612 (48.1%), 1876 (48.1%), 1849 (47.6%), and 1912 (49.2%) for each quintile, respectively.

Abbreviations: BMI, body mass index; COPD, chronic obstructive pulmonary disease; IMD, Index of Multiple Deprivation; IQR, interquartile range.

# **Appendix S5. Age and gender specific incidence rate of dementia**

When compared with the general population from the Cognitive Function and Ageing Studies II [1], the incidence rates of dementia observed in our study approximately agreed with the risk ratios summarised in the meta-analysis, which ranged from 1.42 (95% CI 1.20-1.67) to 3.28 (1.92-5.62) [2].

**Table S5-1. Age and gender specific incidence rates of dementia in comparison with CFAS II**

|  | Stroke patients in the CPRD (N=15 879)  per 1000 person years | General population in the CFAS II (N=5288)  per 1000 person years [1] |
| --- | --- | --- |
| Male (age years) |  |  |
| 65-69 | 8.2 | 5.0 |
| 70-74 | 22.6 | 8.7 |
| 75-79 | 33.2 | 16.7 |
| 80-84 | 49.4 | 24.8 |
| ≥85 | 62.0 | 38.0 |
| Female (age years) |  |  |
| 65-69 | 7.6 | 4.6 |
| 70-74 | 18.7 | 6.4 |
| 75-79 | 35.5 | 16.1 |
| 80-84 | 48.5 | 39.6 |
| ≥85 | 76.6 | 55.3 |

Abbreviation: CFAS, the Cognitive Function and Ageing Studies

Reference:

1. Matthews FE, Stephan BC, Robinson L, Jagger C, Barnes LE, Arthur A, et al. A two decade dementia incidence comparison from the Cognitive Function and Ageing Studies I and II. *Nat Commun*. 2016;7:11398.

2. Kuzma E, Lourida I, Moore SF, Levine DA, Ukoumunne OC, Llewellyn DJ. Stroke and dementia risk: a systematic review and meta-analysis. *Alzheimers Dement*. 2018;14(11):1416-26.

# **Appendix S6. Alternative adjustment strategies for modelling**

In this analysis, we added smaller groups of comorbidities and other blood lipids sequentially in the adjusted Model 2 to examine what were the main confounders affecting the association estimates.

**Table S6-1. Adjusted association between blood lipids (per log-mmol/L increase) and post-stroke dementia in Model 2**

|  | Adjusted hazard ratio (95% confidence interval) | | | |
| --- | --- | --- | --- | --- |
|  | Model 1 | Model 2a | Model 2b | Model 2c |
| LDL cholesterol | 1.08 (0.96-1.22) | 1.21 (1.07-1.37) | 1.20 (1.06-1.36) | 1.26 (1.11-1.43) |
| HDL cholesterol | 0.92 (0.76-1.10) | 1.06 (0.88-1.27) | 1.02 (0.85-1.22) | 0.90 (0.74-1.09) |
| Triglycerides | 0.91 (0.81-1.01) | 0.86 (0.76-0.96) | 0.86 (0.76-0.96) | 0.80 (0.71-0.91) |

Model 1 (the same as Model 1 in Table 2 of the main text): adjusted for age (cubic spline variables), gender, IMD, smoking, and BMI (logarithmic).

Model 2a: adjusted for the variables in Model 1 plus cardiovascular comorbidities/risk factors (stroke subtype, atrial fibrillation, alcohol problem, coronary heart disease, diabetes, heart failure, hypertension, peripheral artery disease and transient ischemic attack).

Model 2b: adjusted for the variables in Model 2a plus other comorbidities (anxiety, rheumatoid arthritis, asthma, chronic obstructive pulmonary disease, depression, epilepsy, hearing loss, and Parkinson’s disease)

Model 2c (the same as Model 2 in Table 2 of the main text): adjusted for the variables in Model 2b plus other two lipid fractions (log-mmol/L).

Abbreviations: BMI, body mass index; HDL, high-density lipoprotein; IMD, Index of Multiple Deprivation; LDL, low-density lipoprotein.

# **Appendix S7. Sensitivity analyses**

**Table S7-1. Sensitivity analysis by using multiple imputation for missing data**

|  | **Adjusted hazard ratio (95% confidence interval)** | | |
| --- | --- | --- | --- |
|  | **LDL cholesterol** | **HDL cholesterol** | **Triglycerides** |
| Per log-mmol/L increase | **1.27 (1.12-1.44)** | 0.89 (0.76-1.05) | **0.80 (0.73-0.89)** |
| **Quintiles for each lipid fraction** | |  |  |
| Q1 | Reference | Reference | Reference |
| Q2 | 1.09 (0.98-1.21) | 0.97 (0.88-1.08) | 0.92 (0.84-1.01) |
| Q3 | **1.15 (1.03-1.29)** | 0.97 (0.87-1.09) | **0.87 (0.79-0.97)** |
| Q4 | **1.19 (1.05-1.34)** | 0.94 (0.84-1.06) | **0.82 (0.73-0.92)** |
| Q5 | **1.32 (1.13-1.53)** | 0.91 (0.79-1.06) | **0.77 (0.67-0.89)** |
| P-value for trend | **<0.001** | 0.20 | **<0.001** |
| **Previous targets for LDL cholesterol** | |  |  |
| <1.8 mmol/L | Reference | NA | NA |
| 1.8-2.59 | **1.11 (1.01-1.22)** | NA | NA |
| 2.60-3.99 | **1.20 (1.08-1.34)** | NA | NA |
| ≥4.0 | **1.33 (1.12-1.57)** | NA | NA |
| P-value for trend | **<0.001** | NA | NA |
| **Current targets for LDL cholesterol** | |  |  |
| <1.4 mmol/L | Reference | NA | NA |
| 1.4-1.79 | 1.06 (0.93-1.21) | NA | NA |
| 1.80-2.59 | **1.15 (1.01-1.31)** | NA | NA |
| 2.6-2.99 | **1.19 (1.01-1.39)** | NA | NA |
| ≥3.0 | **1.32 (1.11-1.56)** | NA | NA |
| P-value for trend | **<0.001** | NA | NA |

All eligible patients (n= 63 959) were included in all the models.

Tests for linear trend were conducted by assigning the medians of each log-lipid fraction to each quintile/target in each imputed dataset and treating the variable as a numerical variable in the Cox models.

All the models adjusted for age (cubic spline variables), gender, IMD, smoking, BMI (logarithmic), comorbidities (stroke subtype, atrial fibrillation, alcohol problem, anxiety, rheumatoid arthritis, asthma, chronic obstructive pulmonary disease, coronary heart disease, depression, diabetes, epilepsy, hearing loss, heart failure, hypertension, Parkinson’s disease, peripheral artery disease and transient ischemic attack), other two lipid fractions (log-mmol/L), consultation (cubic spline variables) and medications (statins, other lipid-lowering drugs, anticoagulant, antiplatelet, antihypertensive drugs, and antidiabetic drugs).

Abbreviations: BMI, body mass index; HDL, high-density lipoprotein; IMD, Index of Multiple Deprivation; LDL, low-density lipoprotein.

**Table S7-2. Sensitivity analysis by starting follow-up from the 4^th^ month after stroke**

|  | **Adjusted hazard ratio (95% confidence interval)** | | |
| --- | --- | --- | --- |
|  | **LDL cholesterol** | **HDL cholesterol** | **Triglycerides** |
| Per log-mmol/L increase | **1.26 (1.10-1.44)** | 0.95 (0.78-1.16) | **0.80 (0.69-0.91)** |
| **Quintiles for each lipid fraction** | |  |  |
| Q1 | Reference | Reference | Reference |
| Q2 | 1.14 (0.98-1.33) | 1.03 (0.87-1.21) | 1.04 (0.90-1.19) |
| Q3 | **1.20 (1.03-1.39)** | 1.03 (0.88-1.21) | 0.90 (0.77-1.04) |
| Q4 | **1.19 (1.02-1.39)** | 1.02 (0.86-1.21) | **0.80 (0.68-0.95)** |
| Q5 | **1.37 (1.17-1.61)** | 0.93 (0.77-1.13) | **0.79 (0.66-0.95)** |
| P-value for trend | **<0.001** | 0.42 | **0.001** |
| **Previous targets for LDL cholesterol** | |  |  |
| <1.8 mmol/L | Reference | NA | NA |
| 1.8-2.59 | **1.16 (1.03-1.32)** | NA | NA |
| 2.60-3.99 | **1.24 (1.08-1.41)** | NA | NA |
| ≥4.0 | **1.30 (1.05-1.59)** | NA | NA |
| P-value for trend | **<0.001** | NA | NA |
| **Current targets for LDL cholesterol** | |  |  |
| <1.4 mmol/L | Reference | NA | NA |
| 1.4-1.79 | 0.98 (0.81-1.19) | NA | NA |
| 1.80-2.59 | 1.15 (0.97-1.37) | NA | NA |
| 2.6-2.99 | 1.06 (0.86-1.32) | NA | NA |
| ≥3.0 | **1.31 (1.09-1.57)** | NA | NA |
| P-value for trend | **<0.001** | NA | NA |

Only those with complete baseline data (n=15 160) were included in all the models.

Tests for linear trend were conducted by assigning the medians of each log-lipid fraction to each quintile/target and treating the variable as a numerical variable in the Cox models.

All the models adjusted for age (cubic spline variables), gender, IMD, smoking, BMI (logarithmic), comorbidities (stroke subtype, atrial fibrillation, alcohol problem, anxiety, rheumatoid arthritis, asthma, chronic obstructive pulmonary disease, coronary heart disease, depression, diabetes, epilepsy, hearing loss, heart failure, hypertension, Parkinson’s disease, peripheral artery disease and transient ischemic attack), other two lipid fractions (log-mmol/L), consultation (cubic spline variables) and medications (statins, other lipid-lowering drugs, anticoagulant, antiplatelet, antihypertensive drugs, and antidiabetic drugs).

Abbreviations: BMI, body mass index; HDL, high-density lipoprotein; IMD, Index of Multiple Deprivation; LDL, low-density lipoprotein.

**Table S7-3. Sensitivity analysis by excluding patients with a first record of dementia within the first 6-month follow-up**

|  | **Adjusted hazard ratio (95% confidence interval)** | | |
| --- | --- | --- | --- |
|  | **LDL cholesterol** | **HDL cholesterol** | **Triglycerides** |
| Per log-mmol/L increase | **1.26 (1.09-1.46)** | 0.96 (0.79-1.18) | **0.77 (0.67-0.89)** |
| **Quintiles for each lipid fraction** | |  |  |
| Q1 | Reference | Reference | Reference |
| Q2 | 1.17 (1.00-1.38) | 1.05 (0.88-1.25) | 1.02 (0.88-1.18) |
| Q3 | **1.24 (1.05-1.45)** | 1.05 (0.90-1.24) | 0.86 (0.73-1.01) |
| Q4 | **1.23 (1.04-1.46)** | 1.05 (0.88-1.25) | **0.78 (0.65-0.93)** |
| Q5 | **1.38 (1.16-1.64)** | 0.96 (0.79-1.17) | **0.77 (0.64-0.92)** |
| P-value for trend | **<0.001** | 0.60 | **<0.001** |
| **Previous targets for LDL cholesterol** | |  |  |
| <1.8 mmol/L | Reference | NA | NA |
| 1.8-2.59 | **1.19 (1.04-1.36)** | NA | NA |
| 2.60-3.99 | **1.25 (1.08-1.44)** | NA | NA |
| ≥4.0 | **1.35 (1.09-1.68)** | NA | NA |
| P-value for trend | **<0.001** | NA | NA |
| **Current targets** |  |  |  |
| <1.4 mmol/L | Reference | NA | NA |
| 1.4-1.79 | 0.95 (0.78-1.16) | NA | NA |
| 1.80-2.59 | 1.16 (0.96-1.39) | NA | NA |
| 2.6-2.99 | 1.07 (0.85-1.35) | NA | NA |
| ≥3.0 | **1.30 (1.07-1.58)** | NA | NA |
| P-value for trend | **<0.001** | NA | NA |

Only those with complete baseline data (n=15 586) were included in all the models.

Tests for linear trend were conducted by assigning the medians of each log-lipid fraction to each quintile/target and treating the variable as a numerical variable in the Cox models.

All the models adjusted for age (cubic spline variables), gender, IMD, smoking, BMI (logarithmic), comorbidities (stroke subtype, atrial fibrillation, alcohol problem, anxiety, rheumatoid arthritis, asthma, chronic obstructive pulmonary disease, coronary heart disease, depression, diabetes, epilepsy, hearing loss, heart failure, hypertension, Parkinson’s disease, peripheral artery disease and transient ischemic attack), other two lipid fractions (log-mmol/L), consultation (cubic spline variables) and medications (statins, other lipid-lowering drugs, anticoagulant, antiplatelet, antihypertensive drugs, and antidiabetic drugs).

Abbreviations: BMI, body mass index; HDL, high-density lipoprotein; IMD, Index of Multiple Deprivation; LDL, low-density lipoprotein.

**Table S7-4. Sensitivity analysis by separating unspecified stroke from ischaemic stroke**

|  | **Adjusted hazard ratio (95% confidence interval)** | | |
| --- | --- | --- | --- |
|  | **LDL cholesterol** | **HDL cholesterol** | **Triglycerides** |
| Per log-mmol/L increase | **1.31 (1.15-1.48)** | 0.89 (0.73-1.08) | **0.78 (0.69-0.89)** |
| **Quintiles for each lipid fraction** | |  |  |
| Q1 | Reference | Reference | Reference |
| Q2 | 1.14 (0.99-1.32) | 1.00 (0.85-1.17) | 1.01 (0.88-1.16) |
| Q3 | **1.19 (1.03-1.38)** | 0.96 (0.82-1.12) | 0.91 (0.78-1.05) |
| Q4 | **1.19 (1.03-1.39)** | 0.96 (0.81-1.13) | **0.76 (0.65-0.90)** |
| Q5 | **1.46 (1.25-1.70)** | 0.90 (0.75-1.08) | **0.79 (0.67-0.94)** |
| P-value for trend | **<0.001** | 0.22 | **<0.001** |
| **Previous targets for LDL cholesterol** | |  |  |
| <1.8 mmol/L | Reference | NA | NA |
| 1.8-2.59 | **1.16 (1.03-1.31)** | NA | NA |
| 2.60-3.99 | **1.27 (1.12-1.44)** | NA | NA |
| ≥4.0 | **1.37 (1.13-1.66)** | NA | NA |
| P-value for trend | **<0.001** | NA | NA |
| **Current targets for LDL cholesterol** | |  |  |
| <1.4 mmol/L | Reference | NA | NA |
| 1.4-1.79 | 0.96 (0.81-1.15) | NA | NA |
| 1.80-2.59 | 1.14 (0.97-1.33) | NA | NA |
| 2.6-2.99 | 1.05 (0.86-1.29) | NA | NA |
| ≥3.0 | **1.35 (1.14-1.60)** | NA | NA |
| P-value for trend | **<0.001** | NA | NA |

Only those with complete baseline data (n=15 879) were included in all the models.

Tests for linear trend were conducted by assigning the medians of each log-lipid fraction to each quintile/target and treating the variable as a numerical variable in the Cox models.

*All the models adjusted for age (cubic spline variables), gender, IMD, smoking, BMI (logarithmic), comorbidities (stroke subtype, atrial fibrillation, alcohol problem, anxiety, rheumatoid arthritis, asthma, chronic obstructive pulmonary disease, coronary heart disease, depression, diabetes, epilepsy, hearing loss, heart failure, hypertension, Parkinson’s disease, peripheral artery disease and transient ischemic attack), other two lipid fractions (log-mmol/L), consultation (cubic spline variables) and medications (statins, other lipid-lowering drugs, anticoagulant, antiplatelet, antihypertensive drugs, and antidiabetic drugs).

Abbreviations: BMI, body mass index; HDL, high-density lipoprotein; IMD, Index of Multiple Deprivation; LDL, low-density lipoprotein.

**Table S7-5. Sensitivity analysis by restricting to patients with linkage to Hospital Episode Statistics**

|  | **Adjusted hazard ratio (95% confidence interval)** | | |
| --- | --- | --- | --- |
|  | **LDL cholesterol** | **HDL cholesterol** | **Triglycerides** |
| Per log-mmol/L increase | **1.31 (1.11-1.54)** | 0.83 (0.66-1.05) | **0.74 (0.63-0.86)** |
| **Quintiles for each lipid fraction** | |  |  |
| Q1 | Reference | Reference | Reference |
| Q2 | 1.15 (0.97-1.37) | 0.90 (0.74-1.08) | 0.97 (0.82-1.15) |
| Q3 | **1.28 (1.08-1.52)** | 0.98 (0.81-1.19) | 0.95 (0.79-1.14) |
| Q4 | **1.31 (1.08-1.58)** | 0.94 (0.78-1.14) | **0.72 (0.59-0.87)** |
| Q5 | **1.39 (1.15-1.68)** | 0.82 (0.66-1.03) | **0.75 (0.61-0.93)** |
| P-value for trend | **<0.001** | 0.12 | **<0.001** |
| **Previous targets for LDL cholesterol** | |  |  |
| <1.8 mmol/L | Reference | NA | NA |
| 1.8-2.59 | **1.21 (1.05-1.40)** | NA | NA |
| 2.60-3.99 | **1.30 (1.10-1.53)** | NA | NA |
| ≥4.0 | **1.38 (1.09-1.74)** | NA | NA |
| P-value for trend | **<0.001** | NA | NA |
| **Current targets for LDL cholesterol** | |  |  |
| <1.4 mmol/L | Reference | NA | NA |
| 1.4-1.79 | 1.04 (0.84-1.30) | NA | NA |
| 1.80-2.59 | **1.24 (1.03-1.51)** | NA | NA |
| 2.6-2.99 | 1.19 (0.93-1.52) | NA | NA |
| ≥3.0 | **1.42 (1.15-1.75)** | NA | NA |
| P-value for trend | **<0.001** | NA | NA |

Only those with complete baseline data (n=8458) were included in all the models.

Tests for linear trend were conducted by assigning the medians of each log-lipid fraction to each quintile/target and treating the variable as a numerical variable in the Cox models.

*All the models adjusted for age (cubic spline variables), gender, IMD, smoking, BMI (logarithmic), comorbidities (stroke subtype, atrial fibrillation, alcohol problem, anxiety, rheumatoid arthritis, asthma, chronic obstructive pulmonary disease, coronary heart disease, depression, diabetes, epilepsy, hearing loss, heart failure, hypertension, Parkinson’s disease, peripheral artery disease and transient ischemic attack), other two lipid fractions (log-mmol/L), consultation (cubic spline variables) and medications (statins, other lipid-lowering drugs, anticoagulant, antiplatelet, antihypertensive drugs, and antidiabetic drugs).

Abbreviations: BMI, body mass index; HDL, high-density lipoprotein; IMD, Index of Multiple Deprivation; LDL, low-density lipoprotein.

**Table S7-6. Sensitivity analysis by using competing-risks regression models**

|  | **Adjusted hazard ratio (95% confidence interval)** | | |
| --- | --- | --- | --- |
|  | **LDL cholesterol** | **HDL cholesterol** | **Triglycerides** |
| Per log-mmol/L increase | **1.27 (1.12-1.45)** | 0.95 (0.79-1.16) | **0.81 (0.72-0.92)** |
| **Quintiles for each lipid fraction** | |  |  |
| Q1 | Reference | Reference | Reference |
| Q2 | 1.14 (0.99-1.32) | 1.04 (0.88-1.22) | 1.03 (0.89-1.18) |
| Q3 | **1.19 (1.03-1.38)** | 1.00 (0.85-1.17) | 0.91 (0.79-1.05) |
| Q4 | **1.18 (1.02-1.38)** | 1.02 (0.86-1.21) | **0.80 (0.69-0.94)** |
| Q5 | **1.43 (1.23-1.66)** | 0.95 (0.79-1.15) | **0.84 (0.71-0.99)** |
| P-value for trend | **<0.001** | 0.56 | **0.003** |
| **Previous targets for LDL cholesterol** | |  |  |
| <1.8 mmol/L | Reference | NA | NA |
| 1.8-2.59 | **1.16 (1.02-1.31)** | NA | NA |
| 2.60-3.99 | **1.26 (1.11-1.42)** | NA | NA |
| ≥4.0 | **1.33 (1.10-1.60)** | NA | NA |
| P-value for trend | **<0.001** | NA | NA |
| **Current targets for LDL cholesterol** | |  |  |
| <1.4 mmol/L | Reference | NA | NA |
| 1.4-1.79 | 0.97 (0.81-1.17) | NA | NA |
| 1.80-2.59 | 1.14 (0.97-1.34) | NA | NA |
| 2.6-2.99 | 1.04 (0.85-1.28) | NA | NA |
| ≥3.0 | **1.34 (1.13-1.59)** | NA | NA |
| P-value for trend | **<0.001** | NA | NA |

Only those with complete baseline data (n=15 879) were included in all the models.

Tests for linear trend were conducted by assigning the medians of each log-lipid fraction to each quintile/target and treating the variable as a numerical variable in the Cox models.

*All the models adjusted for age (cubic spline variables), gender, IMD, smoking, BMI (logarithmic), comorbidities (stroke subtype, atrial fibrillation, alcohol problem, anxiety, rheumatoid arthritis, asthma, chronic obstructive pulmonary disease, coronary heart disease, depression, diabetes, epilepsy, hearing loss, heart failure, hypertension, Parkinson’s disease, peripheral artery disease and transient ischemic attack), other two lipid fractions (log-mmol/L), consultation (cubic spline variables) and medications (statins, other lipid-lowering drugs, anticoagulant, antiplatelet, antihypertensive drugs, and antidiabetic drugs).

Abbreviations: BMI, body mass index; HDL, high-density lipoprotein; IMD, Index of Multiple Deprivation; LDL, low-density lipoprotein.

# **Appendix S8. Subgroup analyses**

**Figure S8-1. Subgroup analysis for LDL cholesterol**

Only those with complete baseline data (n=15 879) were included in all the models.
Tests for linear trend were conducted by assigning the medians of log-LDL cholesterol to each ordinal level for each subgroup and treating the variable as a numerical variable in the Cox models.

The P-value for interaction was obtained by testing the significance of interaction term between the stratifying variable and LDL cholesterol (per log-mmol/L increase).

All the models adjusted for age (cubic spline variables), gender, IMD, smoking, BMI (logarithmic), comorbidities (stroke subtype, atrial fibrillation, alcohol problem, anxiety, rheumatoid arthritis, asthma, chronic obstructive pulmonary disease, coronary heart disease, depression, diabetes, epilepsy, hearing loss, heart failure, hypertension, Parkinson’s disease, peripheral artery disease and transient ischemic attack), other two lipid fractions (log-mmol/L), consultation (cubic spline variables) and medications (statins, other lipid-lowering drugs, anticoagulant, antiplatelet, antihypertensive drugs, and antidiabetic drugs).

Abbreviations: BMI, body mass index; CI, confidence interval; HR, hazard ratio; IMD, Index of Multiple Deprivation; LDL, low-density lipoprotein.

**Figure S8-2. Subgroup analysis for HDL cholesterol**

Only those with complete baseline data (n=15 879) were included in all the models.
Tests for linear trend were conducted by assigning the medians of log-HDL cholesterol to each ordinal level for each subgroup and treating the variable as a numerical variable in the Cox models.

The P-value for interaction was obtained by testing the significance of interaction term between the stratifying variable and HDL cholesterol (per log-mmol/L increase).

All the models adjusted for age (cubic spline variables), gender, IMD, smoking, BMI (logarithmic), comorbidities (stroke subtype, atrial fibrillation, alcohol problem, anxiety, rheumatoid arthritis, asthma, chronic obstructive pulmonary disease, coronary heart disease, depression, diabetes, epilepsy, hearing loss, heart failure, hypertension, Parkinson’s disease, peripheral artery disease and transient ischemic attack), other two lipid fractions (log-mmol/L), consultation (cubic spline variables) and medications (statins, other lipid-lowering drugs, anticoagulant, antiplatelet, antihypertensive drugs, and antidiabetic drugs).

Abbreviations: BMI, body mass index; CI, confidence interval; HDL, high-density lipoprotein; HR, hazard ratio; IMD, Index of Multiple Deprivation.

**Figure S8-3. Subgroup analysis for triglycerides**

Only those with complete baseline data (n=15 879) were included in all the models.
Tests for linear trend were conducted by assigning the medians of log-triglycerides to each ordinal level for each subgroup and treating the variable as a numerical variable in the Cox models.

The P-value for interaction was obtained by testing the significance of interaction term between the stratifying variable and triglycerides (per log-mmol/L increase).

All the models adjusted for age (cubic spline variables), gender, IMD, smoking, BMI (logarithmic), comorbidities (stroke subtype, atrial fibrillation, alcohol problem, anxiety, rheumatoid arthritis, asthma, chronic obstructive pulmonary disease, coronary heart disease, depression, diabetes, epilepsy, hearing loss, heart failure, hypertension, Parkinson’s disease, peripheral artery disease and transient ischemic attack), other two lipid fractions (log-mmol/L), consultation (cubic spline variables) and medications (statins, other lipid-lowering drugs, anticoagulant, antiplatelet, antihypertensive drugs, and antidiabetic drugs).

Abbreviations: BMI, body mass index; CI, confidence interval; HR, hazard ratio; IMD, Index of Multiple Deprivation.

# **Appendix S9. Association of blood lipid fractions with control outcomes**

**Table S9-1. Association of blood lipid fractions (per log-mmol/L increase) with control outcomes**

|  | **LDL cholesterol** | **HDL cholesterol** | **Triglycerides** |
| --- | --- | --- | --- |
| **CHD** |  |  |  |
| Total number | 13 652 | 17 248 | 14 986 |
| Case with CHD | 1505 | 1898 | 1708 |
| Person-years | 126 004 | 159 211 | 138 031 |
| Rate (per 1000 person-years) | 11.9 | 11.9 | 12.4 |
| cHR (95% CI) * | 0.93 (0.82-1.07) | 0.67 (0.57-0.80) | 1.08 (0.97-1.20) |
| aHR (95% CI) Model 1 ^a^ | 1.03 (0.90-1.18) | 0.68 (0.56-0.83) | 1.15 (1.02-1.28) |
| Model 2 ^b^ | 1.15 (0.99-1.33) | 0.73 (0.59-0.91) | 1.00 (0.88-1.14) |
| Model 3 ^c^ | 1.15 (1.00-1.34) | 0.73 (0.59-0.91) | 1.00 (0.87-1.13) |
| **Fracture** |  |  |  |
| Total number | 12 812 | 16 060 | 14 121 |
| Case with fracture | 1304 | 1691 | 1467 |
| Person-years | 119 460 | 149 232 | 131 576 |
| Rate (per 1000 person-years) | 10.9 | 11.3 | 11.1 |
| cHR (95% CI) * | 0.98 (0.84-1.13) | 2.34 (1.95-2.80) | 0.85 (0.75-0.97) |
| aHR (95% CI) Model 1 ^a^ | 0.91 (0.79-1.06) | 1.34 (1.10-1.64) | 1.02 (0.89-1.17) |
| Model 2 ^b^ | 0.90 (0.77-1.04) | 1.43 (1.15-1.78) | 1.08 (0.94-1.25) |
| Model 3 ^c^ | 0.89 (0.77-1.04) | 1.43 (1.15-1.78) | 1.08 (0.93-1.25) |
| **Peptic ulcer** |  |  |  |
| Total number | 16 589 | 20 949 | 18 223 |
| Case with peptic ulcer | 279 | 350 | 315 |
| Person-years | 164 003 | 207 093 | 180 143 |
| Rate (per 1000 person-years) | 1.7 | 1.7 | 1.7 |
| cHR (95% CI) * | 0.83 (0.62-1.09) | 0.68 (0.44-1.04) | 1.26 (0.97-1.63) |
| aHR (95% CI) Model 1 ^a^ | 0.85 (0.63-1.15) | 0.81 (0.49-1.35) | 1.22 (0.93-1.60) |
| Model 2 ^b^ | 0.82 (0.60-1.12) | 0.88 (0.50-1.52) | 1.24 (0.93-1.66) |
| Model 3 ^c^ | 0.80 (0.59-1.10) | 0.88 (0.50-1.53) | 1.27 (0.94-1.70) |

Only those with complete baseline data were included in all the models for the HR estimates: 12 091, 11 453, and 14 841 patients for CHD, fracture and peptic ulcer, respectively.

* The HR was not adjusted for any potential confounders.

a. Model 1: adjusted for age (cubic spline variables), gender, IMD, smoking, and BMI (logarithmic).

b. Model 2: adjusted for the variables in Model 1 plus comorbidities (stroke subtype, atrial fibrillation, alcohol problem, anxiety, rheumatoid arthritis, asthma, chronic obstructive pulmonary disease, coronary heart disease, depression, diabetes, epilepsy, hearing loss, heart failure, hypertension, Parkinson’s disease, peripheral artery disease and transient ischemic attack) and other two lipid fractions (log-mmol/L).

c. Model 3: adjusted for the variables in Model 2 plus consultation (cubic spline variables) and medications (statins, other lipid-lowering drugs, anticoagulant, antiplatelet, antihypertensive drugs, and antidiabetic drugs).

Abbreviations: aHR, adjusted hazard ratio; BMI, body mass index; CHD, coronary heart disease; cHR, crude hazard ratio; HDL, high-density lipoprotein; IMD, Index of Multiple Deprivation; LDL, low-density lipoprotein.

**Table S9-2. Association of different LDL cholesterol levels (quintiles) with control outcomes**

|  | **Q1** ≤ **1.7 mmol/L** | **Q2 1.71-2.12** | **Q3 2.13-2.64** | **Q4 2.65-3.35** | **Q5 ≥3.36** | **P-trend*** |
| --- | --- | --- | --- | --- | --- | --- |
| **CHD** |  |  |  |  |  |  |
| cHR | Reference | 1.16 (0.97-1.37) | 1.02 (0.86-1.22) | 0.97 (0.80-1.17) | 1.00 (0.84-1.19) | 0.44 |
| aHR Model 1^a^ | Reference | 1.18 (0.99-1.41) | 1.07 (0.90-1.27) | 1.03 (0.85-1.25) | 1.11 (0.93-1.33) | 0.59 |
| Model 2 ^b^ | Reference | 1.22 (1.02-1.46) | 1.14 (0.96-1.36) | 1.13 (0.93-1.37) | 1.26 (1.04-1.53) | 0.06 |
| Model 3 ^c^ | Reference | 1.23 (1.03-1.47) | 1.15 (0.97-1.38) | 1.13 (0.93-1.37) | 1.27 (1.05-1.54) | 0.05 |
| **Fracture** |  |  |  |  |  |  |
| cHR | Reference | 0.94 (0.79-1.12) | 0.92 (0.77-1.10) | 0.87 (0.72-1.04) | 1.00 (0.84-1.18) | 0.67 |
| aHR Model 1^a^ | Reference | 0.90 (0.76-1.08) | 0.91 (0.76-1.09) | 0.82 (0.69-0.99) | 0.94 (0.79-1.11) | 0.26 |
| Model 2 ^b^ | Reference | 0.91 (0.76-1.09) | 0.91 (0.75-1.09) | 0.82 (0.68-0.99) | 0.92 (0.77-1.10) | 0.18 |
| Model 3 ^c^ | Reference | 0.91 (0.77-1.09) | 0.91 (0.76-1.09) | 0.81 (0.67-0.98) | 0.91 (0.76-1.10) | 0.16 |
| **Peptic ulcer** |  |  |  |  |  |  |
| cHR | Reference | 0.97 (0.65-1.45) | 1.32 (0.93-1.88) | 0.79 (0.53-1.17) | 0.79 (0.52-1.20) | 0.18 |
| aHR Model 1^a^ | Reference | 0.97 (0.65-1.45) | 1.32 (0.93-1.88) | 0.80 (0.53-1.20) | 0.81 (0.53-1.24) | 0.26 |
| Model 2 ^b^ | Reference | 0.95 (0.63-1.42) | 1.28 (0.89-1.85) | 0.77 (0.50-1.17) | 0.76 (0.48-1.18) | 0.18 |
| Model 3 ^c^ | Reference | 0.95 (0.63-1.43) | 1.28 (0.89-1.84) | 0.75 (0.50-1.15) | 0.73 (0.47-1.16) | 0.15 |

Only those with complete baseline data were included in all the models: 12 091, 11 453, and 14 841 patients for CHD, fracture and peptic ulcer, respectively.

Tests for linear trend were conducted by assigning the medians of log-LDL cholesterol to each quintile and treating the variable as a numerical variable in the Cox models.

a. Model 1: adjusted for age (cubic spline variables), gender, IMD, smoking, and BMI (logarithmic).

b. Model 2: adjusted for the variables in model 1 plus comorbidities (stroke subtype, atrial fibrillation, alcohol problem, anxiety, rheumatoid arthritis, asthma, chronic obstructive pulmonary disease, coronary heart disease, depression, diabetes, epilepsy, hearing loss, heart failure, hypertension, Parkinson’s disease, peripheral artery disease and transient ischemic attack) and other two lipid fractions (log-mmol/L).

c. Model 3: adjusted for the variables in model 2 plus consultation (cubic spline variables) and medications (statins, other lipid-lowering drugs, anticoagulant, antiplatelet, antihypertensive drugs, and antidiabetic drugs).

Abbreviations: BMI, body mass index; CHD, coronary heart disease; aHR, adjusted hazard ratio; cHR, crude hazard ratio; IMD, Index of Multiple Deprivation.

**Table S9-3. Association of different LDL cholesterol levels (previous guideline targets) with control outcomes**

|  | **<1.8 mmol/L** | **1.8-2.59** | **2.6-3.99** | **≥4.0** | **P-trend*** |
| --- | --- | --- | --- | --- | --- |
| **CHD** |  |  |  |  |  |
| cHR | Reference | 1.00 (0.86-1.16) | 0.95 (0.81-1.12) | 0.86 (0.70-1.06) | 0.19 |
| aHR Model 1^a^ | Reference | 1.03 (0.88-1.19) | 1.02 (0.87-1.20) | 0.98 (0.79-1.21) | 0.99 |
| Model 2 ^b^ | Reference | 1.07 (0.92-1.25) | 1.11 (0.94-1.31) | 1.10 (0.89-1.38) | 0.22 |
| Model 3 ^c^ | Reference | 1.08 (0.93-1.26) | 1.11 (0.94-1.32) | 1.11 (0.89-1.38) | 0.21 |
| **Fracture** |  |  |  |  |  |
| cHR | Reference | 0.93 (0.81-1.08) | 0.94 (0.81-1.09) | 0.96 (0.76-1.21) | 0.52 |
| aHR Model 1^a^ | Reference | 0.91 (0.78-1.05) | 0.89 (0.76-1.03) | 0.89 (0.71-1.11) | 0.14 |
| Model 2 ^b^ | Reference | 0.91 (0.78-1.06) | 0.88 (0.75-1.02) | 0.86 (0.68-1.09) | 0.09 |
| Model 3 ^c^ | Reference | 0.91 (0.78-1.06) | 0.87 (0.74-1.02) | 0.85 (0.68-1.08) | 0.08 |
| **Peptic ulcer** |  |  |  |  |  |
| cHR | Reference | 1.29 (0.95-1.75) | 0.82 (0.58-1.16) | 0.87 (0.52-1.45) | 0.15 |
| aHR Model 1^a^ | Reference | 1.29 (0.95-1.75) | 0.83 (0.59-1.19) | 0.91 (0.54-1.53) | 0.23 |
| Model 2 ^b^ | Reference | 1.27 (0.93-1.73) | 0.80 (0.55-1.16) | 0.85 (0.50-1.45) | 0.16 |
| Model 3 ^c^ | Reference | 1.27 (0.93-1.73) | 0.78 (0.54-1.13) | 0.83 (0.48-1.42) | 0.13 |

Only those with complete baseline data were included in all the models: 12 091, 11 453, 11 453, and 14 841 patients for CHD, fracture and peptic ulcer, respectively.

Tests for linear trend were conducted by assigning the medians of log-LDL cholesterol to each quintile and treating the variable as a numerical variable in the Cox models.

a. Model 1: adjusted for age (cubic spline variables), gender, IMD, smoking, and BMI (logarithmic).

b. Model 2: adjusted for the variables in model 1 plus comorbidities (stroke subtype, atrial fibrillation, alcohol problem, anxiety, rheumatoid arthritis, asthma, chronic obstructive pulmonary disease, coronary heart disease, depression, diabetes, epilepsy, hearing loss, heart failure, hypertension, Parkinson’s disease, peripheral artery disease and transient ischemic attack) and other two lipid fractions (log-mmol/L).

c. Model 3: adjusted for the variables in model 2 plus consultation (cubic spline variables) and medications (statins, other lipid-lowering drugs, anticoagulant, antiplatelet, antihypertensive drugs, and antidiabetic drugs).

Abbreviations: BMI, body mass index; CHD, coronary heart disease; aHR, adjusted hazard ratio; cHR, crude hazard ratio; IMD, Index of Multiple Deprivation.

**Table S9-4. Association of different LDL cholesterol levels (current guideline targets) with control outcomes**

|  | **<1.4 mmol/L** | **1.4-1.79** | **1.80-2.59** | **2.6-2.99** | **≥3.0** | **P-trend*** |
| --- | --- | --- | --- | --- | --- | --- |
| **CHD** |  |  |  |  |  |  |
| cHR | Reference | 1.17 (0.94-1.45) | 1.09 (0.89-1.34) | 1.00 (0.78-1.28) | 1.02 (0.83-1.26) | 0.48 |
| aHR Model 1^a^ | Reference | 1.19 (0.95-1.48) | 1.14 (0.93-1.40) | 1.08 (0.84-1.38) | 1.13 (0.92-1.40) | 0.63 |
| Model 2 ^b^ | Reference | 1.21 (0.96-1.51) | 1.20 (0.97-1.48) | 1.17 (0.91-1.51) | **1.27 (1.02-1.58)** | 0.09 |
| Model 3 ^c^ | Reference | 1.22 (0.98-1.53) | 1.22 (0.99-1.50) | 1.18 (0.91-1.52) | **1.29 (1.03-1.60)** | 0.07 |
| **Fracture** |  |  |  |  |  |  |
| cHR | Reference | 1.06 (0.84-1.35) | 0.97 (0.79-1.19) | 0.84 (0.64-1.10) | 1.03 (0.84-1.26) | 0.82 |
| aHR Model 1^a^ | Reference | 1.04 (0.82-1.32) | 0.93 (0.75-1.15) | 0.79 (0.61-1.04) | 0.95 (0.77-1.17) | 0.27 |
| Model 2 ^b^ | Reference | 1.05 (0.82-1.34) | 0.94 (0.76-1.16) | 0.79 (0.61-1.04) | 0.94 (0.76-1.16) | 0.21 |
| Model 3 ^c^ | Reference | 1.06 (0.83-1.35) | 0.95 (0.76-1.17) | 0.79 (0.60-1.04) | 0.94 (0.76-1.16) | 0.19 |
| **Peptic ulcer** |  |  |  |  |  |  |
| cHR | Reference | 0.88 (0.53-1.45) | 1.20 (0.77-1.85) | 0.97 (0.57-1.66) | 0.70 (0.44-1.12) | 0.08 |
| aHR Model 1^a^ | Reference | 0.88 (0.53-1.46) | 1.20 (0.77-1.86) | 0.99 (0.58-1.70) | 0.72 (0.44-1.17) | 0.13 |
| Model 2 ^b^ | Reference | 0.87 (0.52-1.45) | 1.17 (0.75-1.83) | 0.96 (0.56-1.66) | 0.67 (0.41-1.11) | 0.08 |
| Model 3 ^c^ | Reference | 0.87 (0.52-1.45) | 1.17 (0.75-1.82) | 0.94 (0.55-1.62) | 0.65 (0.39-1.07) | 0.06 |

Only those with complete baseline data were included in all the models: 12 091, 11 453, 20 851, and 14 841 patients for CHD, fracture and peptic ulcer, respectively.

Tests for linear trend were conducted by assigning the medians of log-LDL cholesterol to each quintile and treating the variable as a numerical variable in the Cox models.

a. Model 1: adjusted for age (cubic spline variables), gender, IMD, smoking, and BMI (logarithmic).

b. Model 2: adjusted for the variables in model 1 plus comorbidities (stroke subtype, atrial fibrillation, alcohol problem, anxiety, rheumatoid arthritis, asthma, chronic obstructive pulmonary disease, coronary heart disease, depression, diabetes, epilepsy, hearing loss, heart failure, hypertension, Parkinson’s disease, peripheral artery disease and transient ischemic attack) and other two lipid fractions (log-mmol/L).

c. Model 3: adjusted for the variables in model 2 plus consultation (cubic spline variables) and medications (statins, other lipid-lowering drugs, anticoagulant, antiplatelet, antihypertensive drugs, and antidiabetic drugs).

Abbreviations: BMI, body mass index; CHD, coronary heart disease; aHR, adjusted hazard ratio; cHR, crude hazard ratio; IMD, Index of Multiple Deprivation.

**Table S9-5. Association of different HDL cholesterol levels (quintiles) with control outcomes**

|  | **Q1** ≤ **1.01 mmol/L** | **Q2 1.02-1.20** | **Q3 1.21-1.40** | **Q4 1.41-1.70** | **Q5 ≥ 1.71** | **P-trend*** |
| --- | --- | --- | --- | --- | --- | --- |
| **CHD** |  |  |  |  |  |  |
| cHR | Reference | **0.81 (0.70-0.95)** | **0.85 (0.73-0.99)** | **0.77 (0.65-0.90)** | **0.71 (0.60-0.83)** | **<0.001** |
| aHR Model 1^a^ | Reference | **0.80 (0.69-0.94)** | **0.84 (0.72-0.99)** | **0.76 (0.64-0.90)** | **0.72 (0.60-0.86)** | **<0.001** |
| Model 2 ^b^ | Reference | **0.83 (0.71-0.97)** | 0.88 (0.74-1.04) | **0.80 (0.66-0.96)** | **0.77 (0.63-0.94)** | **0.01** |
| Model 3 ^c^ | Reference | **0.83 (0.71-0.98)** | 0.88 (0.75-1.05) | **0.80 (0.66-0.96)** | **0.77 (0.63-0.94)** | **0.01** |
| **Fracture** |  |  |  |  |  |  |
| cHR | Reference | **1.35 (1.10-1.67)** | **1.72 (1.41-2.08)** | **1.95 (1.61-2.37)** | **2.32 (1.93-2.79)** | **<0.001** |
| aHR Model 1^a^ | Reference | 1.21 (0.98-1.50) | **1.35 (1.11-1.65)** | **1.35 (1.10-1.65)** | **1.42 (1.16-1.75)** | **0.001** |
| Model 2 ^b^ | Reference | **1.24 (1.01-1.54)** | **1.42 (1.16-1.74)** | **1.44 (1.17-1.78)** | **1.53 (1.22-1.93)** | **<0.001** |
| Model 3 ^c^ | Reference | **1.25 (1.01-1.55)** | **1.43 (1.17-1.76)** | **1.45 (1.18-1.79)** | **1.54 (1.22-1.93)** | **<0.001** |
| **Peptic ulcer** |  |  |  |  |  |  |
| cHR | Reference | 0.80 (0.54-1.17) | 0.79 (0.53-1.17) | 0.68 (0.47-1.00) | 0.79 (0.54-1.15) | 0.16 |
| aHR Model 1^a^ | Reference | 0.83 (0.57-1.22) | 0.86 (0.58-1.28) | 0.77 (0.51-1.15) | 0.94 (0.61-1.46) | 0.66 |
| Model 2 ^b^ | Reference | 0.86 (0.58-1.27) | 0.90 (0.60-1.35) | 0.82 (0.54-1.25) | 1.02 (0.63-1.66) | 0.96 |
| Model 3 ^c^ | Reference | 0.86 (0.58-1.27) | 0.91 (0.61-1.36) | 0.83 (0.54-1.26) | 1.02 (0.63-1.66) | 0.97 |

Only those with complete baseline data were included in all the models: 12 091, 11 453, 11 453, and 14 841 patients for CHD, fracture and peptic ulcer, respectively.

Tests for linear trend were conducted by assigning the medians of log-HDL cholesterol to each quintile and treating the variable as a numerical variable in the Cox models.

a. Model 1: adjusted for age (cubic spline variables), gender, IMD, smoking, and BMI (logarithmic).

b. Model 2: adjusted for the variables in model 1 plus comorbidities (stroke subtype, atrial fibrillation, alcohol problem, anxiety, rheumatoid arthritis, asthma, chronic obstructive pulmonary disease, coronary heart disease, depression, diabetes, epilepsy, hearing loss, heart failure, hypertension, Parkinson’s disease, peripheral artery disease and transient ischemic attack) and other two lipid fractions (log-mmol/L).

c. Model 3: adjusted for the variables in model 2 plus consultation (cubic spline variables) and medications (statins, other lipid-lowering drugs, anticoagulant, antiplatelet, antihypertensive drugs, and antidiabetic drugs).

Abbreviations: BMI, body mass index; CHD, coronary heart disease; aHR, adjusted hazard ratio; cHR, crude hazard ratio; IMD, Index of Multiple Deprivation.

**Table S9-6. Association of different triglycerides levels (quintiles) with control outcomes**

|  | **Q1** ≤ **0.9 mmol/L** | **Q2 0.91-1.15** | **Q3 1.16-1.49** | **Q4 1.50-1.99** | **Q5 ≥2.0** | **P-trend*** |
| --- | --- | --- | --- | --- | --- | --- |
| **CHD** |  |  |  |  |  |  |
| cHR | Reference | 1.06 (0.90-1.25) | 1.00 (0.86-1.17) | 1.04 (0.89-1.22) | 1.14 (0.98-1.32) | 0.13 |
| aHR Model 1^a^ | Reference | 1.07 (0.91-1.26) | 1.02 (0.86-1.19) | 1.08 (0.92-1.27) | **1.23 (1.05-1.43)** | **0.02** |
| Model 2 ^b^ | Reference | 1.04 (0.88-1.22) | 0.95 (0.81-1.12) | 0.98 (0.83-1.16) | 1.05 (0.88-1.24) | 0.76 |
| Model 3 ^c^ | Reference | 1.04 (0.88-1.23) | 0.96 (0.81-1.13) | 0.98 (0.83-1.16) | 1.04 (0.88-1.24) | 0.82 |
| **Fracture** |  |  |  |  |  |  |
| cHR | Reference | 1.14 (0.95-1.36) | 0.93 (0.77-1.12) | 1.03 (0.87-1.21) | 0.87 (0.73-1.04) | 0.07 |
| aHR Model 1^a^ | Reference | 1.17 (0.97-1.39) | 0.98 (0.80-1.19) | 1.16 (0.98-1.36) | 1.10 (0.91-1.32) | 0.35 |
| Model 2 ^b^ | Reference | **1.21 (1.01-1.45)** | 1.02 (0.84-1.25) | **1.23 (1.04-1.45)** | 1.20 (0.99-1.46) | 0.08 |
| Model 3 ^c^ | Reference | **1.21 (1.01-1.45)** | 1.03 (0.85-1.25) | **1.23 (1.04-1.45)** | 1.20 (0.98-1.46) | 0.08 |
| **Peptic ulcer** |  |  |  |  |  |  |
| cHR | Reference | **1.53 (1.02-2.28)** | **1.51 (1.01-2.26)** | 1.30 (0.89-1.89) | 1.46 (0.96-2.23) | 0.19 |
| aHR Model 1^a^ | Reference | **1.51 (1.01-2.25)** | **1.51 (1.01-2.28)** | 1.27 (0.86-1.87) | 1.40 (0.91-2.18) | 0.30 |
| Model 2 ^b^ | Reference | **1.53 (1.02-2.31)** | **1.53 (1.01-2.30)** | 1.30 (0.86-1.95) | 1.43 (0.90-2.28) | 0.30 |
| Model 3 ^c^ | Reference | **1.55 (1.03-2.33)** | **1.55 (1.02-2.34)** | 1.32 (0.88-1.99) | 1.47 (0.92-2.35) | 0.26 |

Only those with complete baseline data were included in all the models: 12 091, 11 453, 11 453, and 14 841 patients for CHD, fracture and peptic ulcer, respectively.

Tests for linear trend were conducted by assigning the medians of log-triglycerides to each quintile and treating the variable as a numerical variable in the Cox models.

a. Model 1: adjusted for age (cubic spline variables), gender, IMD, smoking, and BMI (logarithmic).

b. Model 2: adjusted for the variables in odel 1 plus comorbidities (stroke subtype, atrial fibrillation, alcohol problem, anxiety, rheumatoid arthritis, asthma, chronic obstructive pulmonary disease, coronary heart disease, depression, diabetes, epilepsy, hearing loss, heart failure, hypertension, Parkinson’s disease, peripheral artery disease and transient ischemic attack) and other two lipid fractions (log-mmol/L).

c. Model 3: adjusted for the variables in Model 2 plus consultation (cubic spline variables) and medications (statins, other lipid-lowering drugs, anticoagulant, antiplatelet, antihypertensive drugs, and antidiabetic drugs).

Abbreviations: BMI, body mass index; CHD, coronary heart disease; aHR, adjusted hazard ratio; cHR, crude hazard ratio; IMD, Index of Multiple Deprivation.
